# Characterization of type-2 diacylglycerol acyltransferases in *Haematococcus lacustris* reveals their functions and engineering potential in triacylglycerol biosynthesis

**DOI:** 10.1186/s12870-020-02794-6

**Published:** 2021-01-06

**Authors:** Hongli Cui, Chunchao Zhao, Wenxin Xu, Hongjiang Zhang, Wei Hang, Xiaoli Zhu, Chunli Ji, Jinai Xue, Chunhui Zhang, Runzhi Li

**Affiliations:** College of Agriculture, Institute of Molecular Agriculture and Bioenergy, Shanxi Agricultural University, Taigu, 030801 Shanxi China

**Keywords:** *Haematococcus lacustris*, Diacylglycerol acyltransferase, Function characterization, Triacylglycerol, Genetic engineering

## Abstract

**Background:**

*Haematococcus lacustris* is an ideal source of astaxanthin (AST), which is stored in oil bodies containing esterified AST (EAST) and triacylglycerol (TAG). Diacylglycerol acyltransferases (DGATs) catalyze the last step of acyl-CoA-dependent TAG biosynthesis and are also considered as crucial enzymes involved in EAST biosynthesis in *H. lacustris.* Previous studies have identified four putative DGAT2-encoding genes in *H. lacustris*, and only HpDGAT2D allowed the recovery of TAG biosynthesis, but the engineering potential of HpDGAT2s in TAG biosynthesis remains ambiguous.

**Results:**

Five putative DGAT2 genes (*HpDGAT2A*, *HpDGAT2B*, *HpDGAT2C*, *HpDGAT2D*, and *HpDGAT2E*) were identified in *H. lacustris*. Transcription analysis showed that the expression levels of the *HpDGAT2A*, *HpDGAT2D*, and *HpDGAT2E* genes markedly increased under high light and nitrogen deficient conditions with distinct patterns, which led to significant TAG and EAST accumulation. Functional complementation demonstrated that HpDGAT2A, HpDGAT2B, HpDGAT2D, and HpDGAT2E had the capacity to restore TAG synthesis in a TAG-deficient yeast strain (H1246) showing a large difference in enzymatic activity. Fatty acid (FA) profile assays revealed that HpDGAT2A, HpDGAT2D, and HpDGAT2E, but not HpDGAT2B, preferred monounsaturated fatty acyl-CoAs (MUFAs) for TAG synthesis in yeast cells, and showed a preference for polyunsaturated fatty acyl-CoAs (PUFAs) based on their feeding strategy. The heterologous expression of *HpDGAT2D* in *Arabidopsis thaliana* and *Chlamydomonas reinhardtii* significantly increased the TAG content and obviously promoted the MUFAs and PUFAs contents.

**Conclusions:**

Our study represents systematic work on the characterization of HpDGAT2s by integrating expression patterns, AST/TAG accumulation, functional complementation, and heterologous expression in yeast, plants, and algae. These results (1) update the gene models of *HpDGAT2s*, (2) prove the TAG biosynthesis capacity of HpDGAT2s, (3) show the strong preference for MUFAs and PUFAs, and (4) offer target genes to modulate TAG biosynthesis by using genetic engineering methods.

**Supplementary Information:**

The online version contains supplementary material available at 10.1186/s12870-020-02794-6.

## Background

Triacylglycerol (TAG) is the principal energy storage form in eukaryotic organisms and represents a promising source of biodiesel production [1]. Microalgae can efficiently absorb CO_2_ in the atmosphere and turn it into abundant high-value products, including polysaccharides, lipids, proteins, pigments, and biofuels [2–5]. Due to their high photosynthetic efficiency, rapid reproduction rate, and short growth cycle, microalgae have been considered as the best candidates to resolve energy crises and environmental pollution [6]. Further understanding of the pathways and regulatory mechanisms involved in TAG accumulation will facilitate the genetic engineering of microalgae [7–9].

Generally, TAG biosynthesis takes place in the endoplasmic reticulum, and TAG assembly can be divided into acyl-CoA-dependent and acyl-CoA independent pathways [10]. Diacylglycerol acyltransferases (DGATs) catalyze the final acylation of sn-1, 2-diacylglycerol (DAG) to form TAG, which is the last and limiting step in the acyl-CoA dependent TAG formation pathway [11]. These enzymes represent a bottleneck in TAG biosynthesis in some oilseed crops and algae, and thus have been regarded as key targets for manipulating TAG production [11]. In higher plants and microalgae, there are four major groups of DGATs: (1) the membrane bound form of DGAT1, (2) the membrane bound form of DGAT2 sharing low sequence similarity with DGAT1, (3) the soluble type of DGAT3, which is localized in the cytosol, and (4) the dual function of WS/DGAT, which possesses both wax ester and TAG biosynthesis activities [12–18]. DGAT1s play a critical role in TAG accumulation in many higher plants and microalgae, whereas DGAT2s appear to have an important role in the formation of TAGs containing unusual fatty acids (FAs) [14]. There is strong evidence supporting the involvement of DGAT3 and WS/DGAT in TAG biosynthesis in microalgae [15, 16]. Usually, only one or two alleles of DGAT1s are identified in a number of microalgae, whereas multiple alleles of DGAT2s are typically present, suggesting that DGAT2s may have an important function in TAG biosynthesis [12–14, 19–27]. Recently, most of the current knowledge about algal DGATs is derived from limited algal species, including *Chlamydomonas reinhardtii*, *Chlorella ellipsoidea*, *Nannochloropsis oceanica*, *Lobosphaera incise*, *Chlorella*/*Chromochloris zofingiensis*, *Myrmecia incise*, and *Phaeodactylum tricornutum*, in which DGATs have been manipulated for molecular cloning, biochemical identification, functional characterization, and to assess their engineering potential for modulating TAG biosynthesis [19–28]. Interestingly, diverse microalgae are prominent candidates for DGATs, and the functions of distinct DGATs are unique or species-specific. Therefore, DGATs in other industrially relevant astaxanthin (AST)-producing algae, such as *Haematococcus lacustris*, have garnered research interest [29].

*H. lacustris* is a green microalga widely known for its ability to synthesize the highest amount of AST (4% dry weight) under stress conditions [29, 30]. Natural AST is a red-coloured keto-carotenoid with strong antioxidant ability and important commercial value [31]. Interstingly, under environmental stress, TAG accumulation is concomitant with AST accumulation, which accumulates after biosynthesis from zeaxanthin and canthaxanthin, and is stored in oil bodies containing esterified AST (EAST) and triacylglycerol (TAG) in *H. lacustris* [32–35]. Moreover, previous studies have indicated that the main forms of EAST include monoester AST (M-AST, 70%) and diester AST (D-AST, 25%) [36–40]. Although the exact mechanisms of stress-induced TAG and AST accumulation in *H. lacustris* are not well understood, several lines of evidence have suggested that the biosynthesis of both compounds appears to be linked through the regulation of oil biosynthetic enzymes at the transcription level [40]. Indeed, the accumulation of AST appears to be dependent on the biosynthesis of FAs and accumulation of TAG [34, 41]. In addition, it has been speculated that certain DGATs are candidate enzymes catalyzing the esterification of AST in *H. pluvialis* [34]. Recently, although four putative type-2 DGATs (*HpDGAT2A*, *HpDGAT2B*, *HpDGAT2D*, and *HpDGAT2E*) were identified from *H. pluvialis* (*lacustris*), and only HpDGAT2D had the capability of to restore TAG biosynthesis in a TAG-deficient yeast strain [42], the engineering potential of DGAT2s in TAG biosynthesis remains ambiguous.

By employing the industrially relevant AST-producing alga *H. lacustris*, in the present study, we present systematic work on the characterization of HpDGAT2s by integrating expression patterns, AST/TAG accumulation, functional complementation, and heterologous expression in yeast, plants, and algae. Five putative *HpDGAT2s* were identified in *H. lacustris*, of which, the transcription levels of *HpDGAT2* genes markedly increased under high light (HL) and nitrogen deficient (ND) conditions with distinct patterns, which led to significant TAG and EAST accumulation. HpDGAT2A, HpDGAT2D, and HpDGAT2E rather than HpDGAT2B had strong TAG biosynthesis activity and preferred monounsaturated fatty acyl-CoAs (MUFAs) and polyunsaturated fatty acyl-CoAs (PUFAs). Overexpression experiments indicated the engineering potential of *HpDGAT2D* in modulating TAG accumulation and FAs composition in algae and plants.

## Results

### Molecular cloning and bioinformatics analysis of *HpDGAT2* genes

Based on the *H. lacustris* transcriptome database [43], five putative *DGAT2* genes were predicted by the BLAST method using other DGAT2s from different algal species (Additional file 1: Table S1) as queries. The full-length mRNA sequences of the five genes were obtained by the rapid amplification of cDNA ends (RACEs) method, and the initiation codon, termination codon, 5′-untranslated region (5′-UTR), 3′-untranslated region (3′-UTR), and poly (A) characteristic tail were determined. Five putative *DGAT2* genes were designed, *HpDGAT2A*, *HpDGAT2B*, *HpDGAT2C*, *HpDGAT2D*, and *HpDGAT2E*, by multiple sequence alignment with *CrDGAT2s*, four of which, *HpDGAT2A*, *HpDGAT2B*, *HpDGAT2D*, and *HpDGAT2E*, contained a full-length open reading frame (ORF), while *HpDGAT2C* was a partial sequence (Additional file 2: Table S2 and Additional file 3: Table S3). Then, the full-length ORFs were cloned and sequenced by PCR with primers (Additional file 4: Table S4), which were renamed and deposited in NCBI GenBank (*HpDGAT2A*: MT875161; *HpDGAT2B*: MT875162; *HpDGAT2C*: MT875163; *HpDGAT2D*: MT875164; *HpDGAT2E*: MT875165). To date, this is the highest dose of DGAT2s reported in the green alga *H. lacustris*. Based on a comparison with gene models of *HpDGAT2s* reported by Nguyen et al. [42], our results confirmed that there were five *HpDGAT2s* members in *H. lacustris*. Generally, only one or two alleles of DGAT1s are identified in a number of microalgae, whereas multiple alleles of *DGAT2s* are typically present [14].

To gain insights into the biochemical characteristics of HpDGAT2s, the molecular weight (MW), isoelectric point (pI), subcellular location, transmembrane domain (TM), signal peptide (SP), chloroplast transfer peptide (CTP), and phosphorylation site (Phos) were analyzed. No SP or CTP was present in HpDGAT2s protein sequences except for CTP in HpDGAT2C (Additional file 2: Table S2). There were two TMs in all pDGAT2s protein sequences except for three TMs in HpDGAT2B (Additional file 2: Table S2 and Additional file 5: Fig. S1), which is consistent with the membrane bound forms of DGAT1 and DGAT2 [14]. In addition, 14–30 phosphorylation sites were predicted in HpDGAT2 protein sequences (Additional file 2: Table S2 and Additional file 6: Fig. S2), indicating that phosphorylation plays important roles in DGAT2 enzyme activity because DGAT1 enzyme activity is affected by serine phosphorylation sites in mouse DGAT1 [44], TmDGAT1 [45], and BnDGAT1 [46]. It remains to be determined whether these phosphorylation sites are important for the functional regulation of HpDGAT2 in vivo.

To further analyze the conserved domains (CDs) and evolutionary relationship between HpDGAT2s and other algal DGAT2s, multiple sequence alignment and a phylogenetic tree were reconstructed. CDs analysis showed that HaeDGAT2s contained 7 CDs [26, 47, 48], including YF/YFP block (CD1), which is essential for DGAT2 activity; HPHG/EPHS block (CD2), which is proposed to partially consist of the active site; PxxR (x = random amino acid) block (CD3); xGGxAE block (CD4); RxGFx(K/R)xAxxxGxx(L/V) VPxxxFG block (CD5), which is the longest conserved sequence in plants and animals; PxxxVVGxPIxVP block (CD6); and RHK block (CD7) (Additional file 7: Fig. S3). As shown in Additional file 7: Fig. S3, there were two completely conserved amino acid residues (proline, P and phenylalanine, F) among all DGAT2s, which is consistent with previous reports indicating that these two highly conserved residues may be located at the active sites of the enzymes and make significant contributions to their enzymatic activities [49]. The phylogenetic analysis of the HpDGAT2s and other DGAT orthologues from eukaryotic algae and plants is illustrated in Additional file 8: Fig. S4, which is consistent with most previous results [20–26]. Briefly, all HpDGAT2s clustered with the algal DGAT2s orthologues, which are distinct from other DGAT subfamilies, including DGAT1, DGAT3, and DGAT/WSD. Of the five HpDGAT2s, HpDGAT2A formed a monophyletic subgroup (BS: 100%) with CrDGAT2A, CzDGAT2A, CzDGAT2B, LiDGAT2A, and LiDGAT2B. HpDGAT2B and HpDGAT2E were highly close (BS: 98%) to CrDGAT2B, CzDGAT2E and CrDGAT2C. HpDGAT2C was evolutionarily close (BS: 100%) to CzDGAT2C and LiDGAT2C. HpDGAT2D built a monophyletic subgroup (BS: 73%) with CrDGAT2D and CzDGAT2D.

### AST and TAG accumulation and *HpDGAT2s* gene transcription upon exposure to high light and nitrogen deficient stresses

**Fig. 1:**
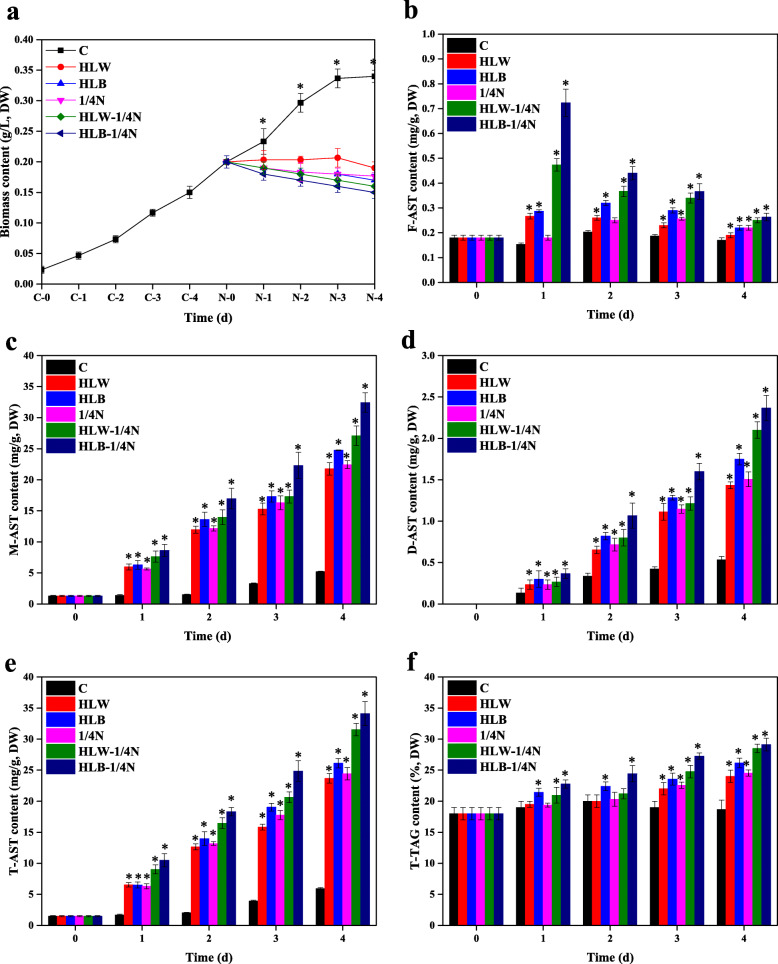
Growth, astaxanthin, and triacylglycerol profiles of *Haematococcus lacustris* under HLW, HLB, 1/4 N, HLW-1/4 N, and HLB-1/4 N conditions after 1, 2, 3, and 4 day. **a** Time course of biomass content. **b** Free astaxanthin content. **c** Monoesterrified astaxanthin content. **d** Diesterrified astaxanthin content. **e** Total astaxanthin content. **f** Total triacylglycerol content

High light (HL) and nitrogen deficient (nitrogen-free, ND) stresses can effectively promote the accumulation of AST and TAG in *H. lacustris* [32–34, 50–53]. However, under such circumstances, the growth of algae was completely restricted [51–53]. Recently, our team completed research investigating the effects of nitrogen deficiency (nitrogen content compared to growth in control BBM medium, e.g., 0, 1/4 N, 1/2 N, and 3/4 N) on algal growth and AST and TAG accumulation. The results indicated that the highest AST productivity was achieved under 1/4 N stress due to a certain level of algal growth. Therefore, in the current manuscript, the 1/4 N condition was selected as the nitrogen deficient stress for further experiments. To understand the relationship between *HpDGAT2s* transcription and TAG and AST biosynthesis, time-course patterns of algal biomass, expression, total AST (T-AST), and total TAG (T-TAG) contents in photoautotrophic cultures of *H. lacustris* under HL, 1/4 N, and double HL-1/4 N stresses were studied (Fig. 1).

**Fig. 2:**
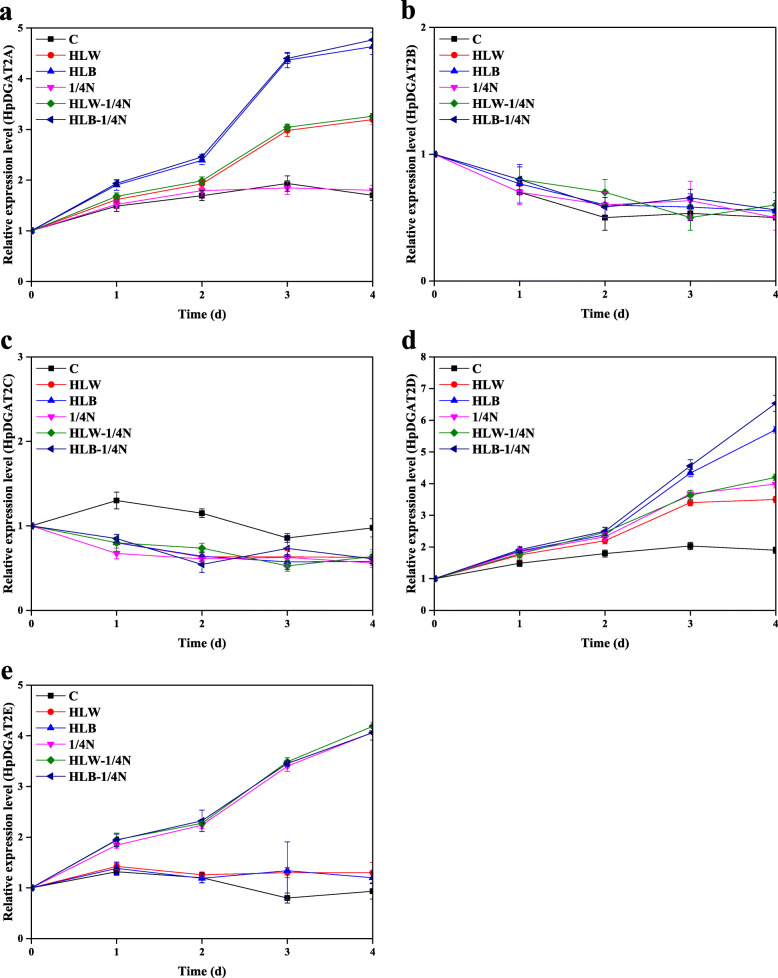
The transcriptional expression levels of *HpDGAT2s* in *Haematococcus lacustris* under HLW, HLB, 1/4 N, HLW-1/4 N, and HLB-1/4 N conditions after 1, 2, 3, and 4 day. **a** *HpDGAT2A*. **b** *HpDGAT2B*. **c** *HpDGAT2C*. **d** *HpDGAT2D*. e *HpDGAT2E*. The gene expression levels were normalized to the endogenous *actin* gene

As shown in Fig. 1a, compared to the control, HL, 1/4 N, and double HL-1/4 N stresses inhibited algal growth. The T-AST production and composition are summarized in Fig. 1b-e. From these results, we could draw the conclusions that (1) M-AST is the main form; (2) compared to 1/4 N stress, HL is more effective at inducing AST accumulation, especially under high blue light (HLB) conditions; and (3) coupled HL and 1/4 N dual stimulation might be a better choice for improving AST accumulation. Moreover, T-TAG contents slowly increased from day 1 to day 4 and reached maximum values of 29.5, 28.7, 26.8, 25.2, and 24.8% under HLB-1/4 N, HLW-1/4 N, HLB, 1/4 N, and HLW conditions, respectively, which were 159.5, 155.1, 144.9, 136.2, and 134.1% higher than the values of the control (Fig. 1f). The effects of HL, 1/4 N and double HL-1/4 N stresses on TAG and AST accumulation were largely consistent with previous studies showing that AST and lipid biosynthesis were enhanced and that the former was coordinated with later biosynthesis under HL and ND conditions [34, 41]. Previous studies have indicated that DGAT enzymes are probably responsible for both AST esterification and TAG biosynthesis in *H. lacustris* [33, 34]. As revealed by qRT-PCR results (Fig. 2), the HpDGAT2 gene transcription expression levels exhibited distinct patterns under HL, 1/4 N and double HL-1/4 N stresses. Of the five *HpDGAT2s*, the *HpDGAT2B* and *HpDGAT2C* expression levels decreased and remained constant (Fig. 2b and c). The *HpDGAT2A* and *HpDGAT2E* expression levels increased and reached their maximum at 4 d of exposure, and they were HL and 1/4 N stress-dependent (Fig. 2a and e), respectively, while the *HpDGAT2D* expression level increased and was stress dependent (Fig. 2d). These results suggested that these *HpDGAT2A*, *HpDGAT2D*, and *HpDGAT2E* genes were together involved in AST and TAG biosynthesis under stress.

### Functional complementation of HpDGAT2s in yeast

To verify the function of the putative HpDGAT2s enzymes, the ORF-encoding sequences were cloned (Additional file 4: Table S4) into the pYES2.0 plasmid and heterologously expressed in the quadruple mutant yeast strain *S. cerevisiae* H1246 (*∆dga1∆lro1∆are1∆are2*), which lacks TAG synthesis activity. Mutant type (H1246) yeast can form TAG when at least one of these four genes is expressed. Furthermore, wild-type (INVSc1) and H1246-EV (H1246 harbouring empty vector pYES2.0) yeast strains were used as positive and negative controls, respectively.

**Fig. 3:**
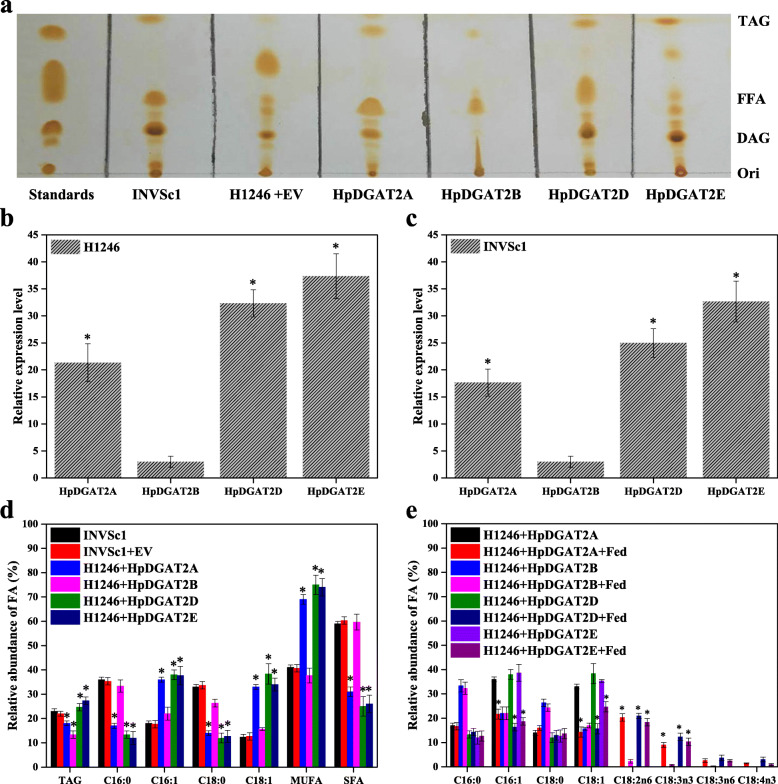
Functional characterization of *HpDGAT2s* in *Saccharomyces cerevisiae* cells. **a** TLC analysis of total lipids extracted from the control *S. cerevisiae* (INVSc1), TAG-deficient *S. cerevisiae* (H1246), and H1246 cells transformed with *HpDGAT2s* and Empty vector (EV). **b** and **c** The transcriptional expression levels of *HpDGAT2s* in H1246 and INVSc1 cells transformed with *HpDGAT2s*. The gene expression levels were normalized to the endogenous *ACT1* gene. **d** TAG contents and relative abundance of fatty acid in INVSc1 and H1246 cells transformed with *HpDGAT2s*. **e** Relative abundance of fatty acid in H1246 cells transformed with *HpDGAT2s* by feeding of free fatty acid of C18:2n6, C18:3n3, C18:3n6, and C18:4n3 after a 24 h cultivation

The expression of *HpDGAT2A*, *HpDGAT2B*, *HpDGAT2D*, and *HpDGAT2E* restored TAG biosynthesis at different levels in H1246 cells, as indicated by the remarkable TAG spot on a TLC plate (Fig. 3a). In contrast, *HpDGAT2B* expression in H1246 cells produced inconspicuous TAG levels, indicating a nonfunctional encoded protein considering the low transcription expression levels in H1246 cells (Fig. 3b) and *H. lacustris* cells (Fig. 2b). Nevertheless, the limited FA composition in *Saccharomyces cerevisiae* might lead to low TAG content for HpDGAT2B. The ability of HpDGAT2A, HpDGAT2B, HpDGAT2D, and HpDGAT2E to restore TAG biosynthesis in yeast led us to examine FA substrate specificity. As indicated in Fig. 3b and c, the *HpDGAT2A*, *HpDGAT2B*, *HpDGAT2D*, and *HpDGAT2E* genes were heterologously expressed in H1246 and INVSc1 cells. The changes in TAG content and FA composition of TAGs extracted from the transformed H1246 and INVSc1 cells were similar. As shown in Fig. 3d, the TAG contents of expressed *HpDGAT2A* and *HpDGAT2B* in H1246 cells were 78.3 and 56.5% lower, respectively, than those of the control (INVSc1 and INVSc1 + EV). The TAG contents of expressed *HpDGAT2D* and *HpDGAT2E* were 108.7 and 122.7% higher, respectively, than the control. To further test FA substrate specificity, FAs from transformed H1246 and INVSc1 cells were analyzed by GC. As shown in Fig. 3d, compared to the control, the MUFAs palmitoleic acid (C16:1) and oleic acid (C18:1) abundances increased in *HpDGAT2A-*, *HpDGAT2D-*, and *HpDGAT2E*-expressing H1246 cells at the expense of saturated fatty acids (SFAs), including palmitic acid (C16:0) and stearic acid (C18:0). Such a tendency, however, at different levels was observed for almost all transformed lines of H1246 for various DGAT enzymes [20, 23–28].

Considering the limited FA composition in yeast strains (C16:0, C18:0, C16:1, and C18:1), some PUFAs enriched in *H. lacustris*, including linoleic acid (C18:2n6), *α*-linolenic acid (C18:3n3), *γ*-linolenic acid (C18:3n6), and parinaric acid (C18:4n3), were tested for substrate specificity for the HpDGAT2A, HpDGAT2B, HpDGAT2D, and HpDGAT2E enzymes by employing a feeding strategy. HpDGAT2A, HpDGAT2D, and HpDGAT2E had similar tendencies to incorporate these PUFAs into TAG at the expense of C16:1 and C18:1 with the following patterns: C18:2n6 > C18:3n3 > C18:3n6 > C18:4n3 (Fig. 3e). Considering that C18:2n6 and C18:3n3 were rich in *H. lacustris*, it is reasonable to speculate that these HpDGAT2s may have potential in C18:2n6- and C18:3n3-enriched TAG production [32–34]. The HpDGAT2A, HpDGAT2D, and HpDGAT2E enzymes showed a stronger preference for PUFAs than MUFAs due to the higher feeding content of PUFAs than endogenous MUFAs content. This phenomenon was also confirmed by Zienkiewicz et al. (2018), who incorporated some PUFAs into TAG at the expense of 16:1 and 18:1 in *LiDGAT1-*, *LiDGAT2.1-*, *LiDGAT2.2-*, and *LiDGAT2.3*-expressing yeast [23] and in *CzDGAT2C-*expressing mutant H1246 yeast cells [26] by feeding tests. However, FA profiles of the TAG fraction from yeast cells expressing *HpDGAT2B* showed no obvious changes, implying a nonfunctional protein (Fig. 3e).

### *HpDGAT2D* heterologous expression promotes TAG biosynthesis and its relative MUFAs and PUFAs abundance in *C. reinhardtii*

To investigate the possible biological role of HpDGAT2s and their engineering potential to modulate TAG biosynthesis in algae, we generated *HpDGAT2D* heterologous expression lines in the evolutionarily close green alga *C. reinhardtii* CC849. *HpDGAT2D* was selected for further experiments due to the relatively strong TAG biosynthetic activity in yeast cells (Fig. 3) and high transcription expression level in *H. lacustris* under stress conditions (Fig. 2d).

**Fig. 4:**
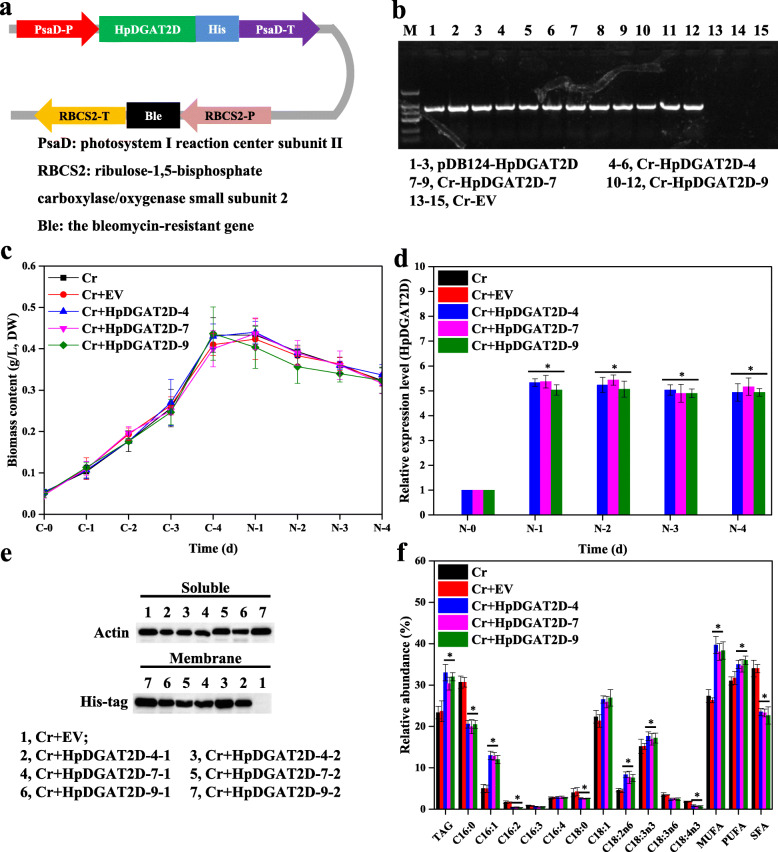
Heterologous expression of *HpDGAT2D* in *Chlamydomonas reinhardtii* cells. **a** Construct of the expression vector. PsaD-P, promoter of *PsaD* gene; His tag, 6-His encoding gene; PsaD-T, terminator of *PsaD* gene; RBCS2-P, promoter of *RBCS2* gene; Ble, the *bleomycin*-resistant gene; RBCS-T, terminator of *RBCS* gene. **b** Genomic level detection of *HpDGAT2D* in *C. reinhardtii* cells. **c** Time course of biomass content under control and 1/4 N conditions. **d** The transcriptional expression levels of *HpDGAT2D* in *C. reinhardtii* cells. The gene expression levels were normalized to the endogenous *actin* gene. **e** Western blotting of HpDGAT2D-His tag fusion protein with His-tagged antibodies. Soluble and membrane proteins were separated and used for blotting. Actin which was known soluble protein was used as controls. **f** TAG contents and relative abundance of fatty acid in *C. reinhardtii* cells transformed with *HpDGAT2D*

The nuclear transformation expression vector pDB124 (Additional file 9: Fig. S5), characterized in *C. reinhardtii* CC849 and gifted by professor Zhangli Hu from Shenzhen University, was used in this study after modification because it contained overexpression cassettes of the *HpDGAT2D-His* fusion and bleomycin resistance *Ble* genes under the control of the verified endogenous promoter and terminator of the *PsaD* and *RBCS2* genes, respectively (Fig. 4a). The codon preference (*HpDGAT2D*) was optimized according to the alga *C. reinhardtii* (Additional file 10: Fig. S6) before constructing the expression vector. Transformants (screening over 20 putative transformants) were selected on TAP plates supplemented with bleomycin and confirmed by genomic PCR. The exogenous *HpDGAT2D-His* fusion gene was integrated into the alga chromosome due to the clear band using the *HpDGAT2D-Cr* gene as primers in transformation lines, whereas no signal was detected in WT cells (Fig. 4b and Additional file 11: Fig. S7a). Three heterologous expression lines, *HpDGAT2D-4*, *HpDGAT2D-7*, and *HpDGAT2D-9*, exhibited a maximum increase in transcription levels (by ~ 5.5-fold higher than the control) under ND conditions in a 4-day batch culture, with no significant difference in cell growth between the transgenic lines and the control (Fig. 4c and d). Furthermore, in vivo heterologous expression of the HpDGAT2D protein was validated by using His-tagged antibodies via western blot analysis. Bands were present in the membrane proteins of three heterologous expression lines (*HpDGAT2D-4*, *HpDGAT2D-7*, and *HpDGAT2D-9*), but were absent from the soluble proteins, which was consistent with HpDGAT2D being a transmembrane enzyme (Fig. 4e and Additional file 11: Fig. S7b). *HpDGAT2D* heterologous expression led to considerable increases (by ~ 1.4-fold) in TAG content under ND conditions (Fig. 4f). *HpDGAT2D* heterologous expression also affected the FA profiles in TAGs (Fig. 4f). A significant increase was observed in the relative abundance of MUFAs (C16:1 and C18:1) and PUFAs (C18:2n6 and C18:3n3), accompanied by a significant decrease in SFAs (C16:0 and C18:0) and some PUFAs (C16:2, C16:3, C18:3n6, and C18:4n3). These results indicated that (1) HpDGAT2D showed a stronger preference for MUFAs and PUFAs than SFAs; (2) of all PUFAs, HpDGAT2D chose C18:2n6 and C18:3n3 as the first option rather than C16:2, C16:3, C18:3n6, and C18:4n3; and (3) these preferred substrates were enriched in *C. reinhardtii*. This trend was consistent with results from yeast cells obtained by feeding test (Fig. 3d and e) and previous studies of *NoDGAT1A* expression in *C. reinhardtii* UVM4 and *CzDGAT1A* expression in oleaginous alga *N. oceanica* by Wei et al. (2017) and Mao et al. (2019), respectively [20, 22].

### *HpDGAT2D* heterologous expression enhances seed oil content and its relative MUFAs and PUFAs abundance in *A. thaliana*

**Fig. 5:**
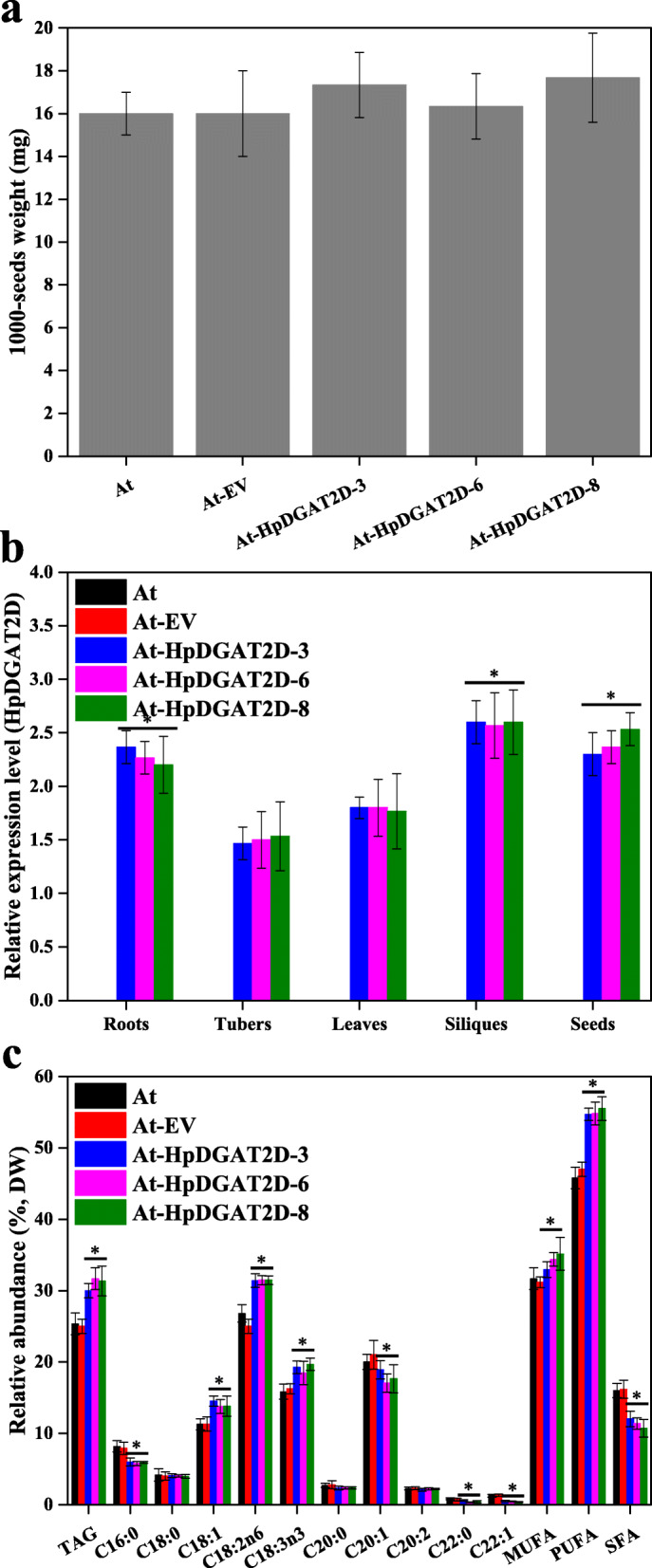
Heterologous expression of *HpDGAT2D* in *Arabidopsis thaliana*. **a** Average 1000-seed weight (expressed as milligrams of weight/1000 seeds) of transgenic *Arabidopsis* T2 seeds. **b** The transcriptional expression levels of *HpDGAT2D* in *A. thaliana*. The gene expression levels were normalized to the endogenous *actin* gene. **c** TAG contents and relative abundance of fatty acid in *A. thaliana* transformed with *HpDGAT2D*

To explore HpDGAT2s as a tool to manipulate acyl-CoA pools and to engineer TAG biosynthesis in higher plants, *HpDGAT2D* was heterologously expressed in *Arabidopsis thaliana*. Three *A. thaliana* independent expression T2 generation lines (*At-HpDGAT2D-3*, *At-HpDGAT2D-6*, and *At-HpDGAT2D-8*) were selected for further detailed analysis. There were no visible morphological difference (e.g., 1000-seed weight) between the transgenic lines and untransformed control *A. thaliana* (Fig. 5a). The qRT-PCR results showed that the *HpDGAT2D* transcript was expressed in transgenic lines in different tissue organs, including roots, tubers, leaves, siliques, and seeds, to different extents (Fig. 5b). The transformation of wild-type *A. thaliana* with *HpDGAT2D* resulted in higher (120.0–126.4%) seed TAG content than the control (Fig. 5c). Again, further GC analysis of FA profiles from TAGs revealed that PUFAs and MUFAs significantly increased, accompanied by a significant decrease in SFAs (Fig. 5c). However, the exact alteration process was much more complicated than those in yeast and *C. reinhardtii* cells. Specifically, of the SFAs, C16:0 and C22:0 decreased while C18:0 and C20:0 remained stable. Of MUFAs and PUFAs, HpDGAT2D preferred C18:1, C18:2n6, and C18:3n3 rather than C20:1, C20:2 and C22:1 in TAG biosynthesis. These results were largely in agreement with those from yeast cells (Fig. 3d and e) and *C. reinhardtii* cells (Fig. 4c). Guo et al. (2017) indicated that the *CeDGAT1* gene can stimulate FA biosynthesis and enhance seed weight and oil content when expressed in *A. thaliana* and *B. napus* [21].

## Discussion

Usually, the accumulation of AST and TAG is simultaneously significantly enhanced under most stress conditions in *H. lacustris*, e.g., HL and ND conditions [29–35, 50–53]. However, in general, nitrogen deficiency seriously limits algal growth [51–53]. Recently, our results indicated that the highest AST productivity was achieved under 1/4 N stress based on a certain level of algal growth. Therefore, in the current manuscript, the 1/4 N condition was selected as the ND condition in further experiments. Our results revealed that (1) T-AST and T-TAG contents significantly increased under HL and 1/4 N conditions, respectively, which was consistent with some previous studies [34, 41]; (2) M-AST was the main form, which has also been proven by previous studies [36–39]; (3) compared to 1/4 N stress, HL was more effective in inducing AST accumulation, especially under high blue light conditions, which was demonstrated in our previous study [50]; and (4) coupled HL and 1/4 N dual stimulation might be better choices for AST and TAG accumulation in *H. lacustris* (Fig. 1) [53]. Although the specific mechanisms of stress-induced TAG and AST accumulation in *H. lacustris* are largely unknown, several lines of evidence have implied that the biosynthesis of TAG and AST appears to be linked to the regulation of oil biosynthetic enzymes at the transcription level [34, 41]. In fact, AST accumulation is dependent on FA biosynthesis and TAG accumulation in *H. lacustris* [34, 41]. Recently, Zhang et al. (2019) reported that synthesized AST was esterified mainly with the fatty acid C18:1 and stored in TAG-filled lipid droplets in *C. zofingiensis* [40]. Unlike in *H. lacustris*, although AST accumulated in a well-coordinated manner with TAG, AST is ketolated from zeaxanthin and is independent of FA synthesis in *C. zofingiensis* [40]. This contrasting result may be due to the differences in the genetic traits of these two organisms. The enzymes involved in EAST biosynthesis in the AST-producing algae *H. lacustris* and *C. zofingiensis* are unclear.

DGATs catalyze the terminal step in the acyl-CoA-dependent TAG production pathway and represent key targets for manipulating TAG production [11]. At present, DGATs from different algal species have been widely studied, which indicates that diverse microalgae are prominent candidates for DGATs and that the function of distinct DGATs is unique or species-specific [19–28]. Obviously, the *HpDGAT2* genes were differentially regulated by HL, 1/4 N, and double HL-1/4 N stress conditions with distinct patterns, suggesting that these enzymes are together involved in AST and TAG biosynthesis (Fig. 2). Mao et al. (2019) indicated that *CzDGAT1A*, *CzDGTT1*, *CzDGTT5* and *CzDGTT8* were all considerably up-regulated by ND with distinct expression patterns [20]. Chen et al. (2015) indicated that the transcript level of *MiDGAT2A* was regulated by ND stress, which led to TAG accumulation [28]. In addition, previous studies have indicated that DGATs are possible candidate enzymes involved in both TAG and EAST accumulation [34], which makes it more interesting to identify DGATs in the AST-producing industrial alga *H. lacustris* [29]. Recently, although four putative type-2 DGAT genes were identified from *H. pluvialis* (*lacustris*), and only HpDGAT2D had the ability to restore TAG biosynthesis in a TAG-deficient yeast strain [42], the engineering potential of DGAT2s in TAG biosynthesis remains ambiguous.

In this study, we demonstrated that there were five DGAT2s genes in the alga *H. lacustris*, which we renamed *HpDGAT2A*, *HpDGAT2B*, *HpDGAT2C*, *HpDGAT2D*, and *HpDGAT2E* according to sequence alignment and phylogenetic analysis results (Additional file 3: Table S3 and Additional file 8: Fig. S4), updating a previous report of four putative type-2 DGATs in the *H. pluvialis* (*lacustris*) transcriptome database [42]. Generally, only one or two copies of DGAT1s are present in a number of microalgae, whereas multiple copies of DGAT2s are typically present [14]. The number of DGAT2s is species-specific in various algal organisms, e.g., *Chlamydomonas reinhardtii* (5), *Nannochloropsis oceanica* (13), *Lobosphaera incise* (3), *Chlorella zofingiensis* (8), *Myrmecia incise* (2), and *Phaeodactylum tricornutum* (4) [20, 23, 24, 26–28]. Subcellular localization prediction revealed the different sub-locations of HpDGAT2s (Additional file 2: Table S2), which is consistent with the subcellular localization prediction of DGATs from the green algae *C. reinhardtii* [24] and *C. zofingiensis* [20]. Two or three TMs were present in all HpDGAT2s (Additional file 2: Table S2 and Additional file 5: Fig. S1), implying they were members of the membrane-bound forms of DGAT1 and DGAT2 [14]. Interestingly, abundant phosphorylation sites were predicted in all HpDGAT2s (Additional file 2: Table S2 and Additional file 6: Fig. S2), indicating that phosphorylation plays important roles in DGAT2s enzyme activity, given that DGAT1 enzyme activity is affected by phosphorylation of mouse DGAT1 [44], BnaDGAT1 [46] and TmaDGAT1 [45]. It remains to be determined whether these phosphorylation sites are important for the functional regulation of HpDGAT2 in vivo. The CDs previously identified in DGAT2 enzymes from higher plants and microalgae [26, 47, 48] were also present in HpDGAT2s but with varying degrees of conservation (Additional file 7: Fig. S3), including YF/YFP block (CD1), which is essential for DGAT2 activity; HPHG/EPHS block (CD4), which is proposed to partially consist of the active site; and RxGFx(K/R)xAxxxGxx(L/V) VPxxxFG block (CD5), which is the longest conserved sequence in plants and animals. Some putative lipid binding motifs (FLxLxxx and FVLF blocks) in mouse DGAT2 were not conserved among HpDGAT2s and algal DGAT2s [47, 48, 54]. Moreover, there were two completely conserved amino acid residues (proline, P and phenylalanine, F) among all DGAT2s, which is consistent with previous reports that these two highly conserved residues may be located at the active sites of the enzymes [49].

To characterize the roles of HpDGAT2s, four *HpDGAT2s* genes with full-length coding sequences (Additional file 2: Table S2) were heterologously expressed in the TAG-deficient yeast strain H1246 [55]. The results indicated that all of the *HpDGAT2s* genes are functional with the large differences in enzymatic activity (Fig. 3a). Further functional characterization in yeast showed that HpDGAT2D and HpDGAT2E can increase the TAG content more than HpDGAT2A and HpDGAT2B, resulting in a significant increase in the TAG content of yeast by 108.7–122.7% (Fig. 3d). This higher activity provides an alternative candidate for DGAT2 to modulate TAG accumulation in algae. However, a previous study detected that only HpDGAT2D had the ability to restore TAG biosynthesis in a TAG-deficient yeast strain [42]. In contrast, in our study, *HpDGAT2B* expression in H1246 cells produced inconspicuous TAG, possibly due to the limited FAs in *Saccharomyces cerevisiae*. This holds true, at least for *CzDGTT1* expressed in yeast, as the TAG content increased when feeding on two other free FAs [20]. It is also possible that HpDGAT2B may not be a real DGAT but another type of transferase, which cannot be differentiated based only on the protein sequence [20]. This phenomenon is usually present in green algae, e.g., CrDGTT1 through CrDGTT3 are functional, while CrDGTT4 is not [24]. NoDGAT1A and CzDGTT1, rather than NoDGAT1B, are functional [20, 22].

DAGs and fatty acyl-CoAs are essential substrates for TAG biosynthesis under the catalysis of DGAT enzymes [20, 22, 24]. The fatty acyl-CoA substrate specificity was determined by FA profile analysis. HpDGAT2s showed a strong preference for MUFAs (C16:1 and C18:1) in yeast cells. Such a tendency, however, at different levels was observed for almost all transformed lines of H1246 for various DGAT enzymes [20, 23, 24, 26–28]. Considering the limited FAs in yeast cells, some PUFAs (e.g., C18:2n6, C18:3n3, C18:3n6, and C18:4n3) that are present in *H. lacustris* but not in yeast cells were selected to test the acyl-CoA substrate specificity by using a feeding strategy. Interestingly, all HpDGAT2s except for HpDGAT2B showed a wide range of preference for PUFAs with distinct patterns in yeast cells, especially for C18:2n6 and C18:3n3, which are also rich in *H. lacustris*, indicating that these HpDGAT2s may have potential for the engineering of PUFAs-enriched TAG production. This phenomenon was also confirmed by Zienkiewicz et al. (2018), who incorporated some PUFAs into TAG at the expense of C16:1 and C18:1 in *LiDGAT1-*, *LiDGAT2.1-*, *LiDGAT2.2-*, and *LiDGAT2.3*-expressing yeast [23] and *CzDGAT2C*-expressing yeast mutant H1246 cells [26] by feeding tests. Consistent with the low transcription of *HpDGAT2B* in algal and yeast cells, the feeding test demonstrated the low preference of PUFAs, again indicating a nonfunctional encoded protein. Although the acyl-CoA substrate preference was characterized, the DAG (prokaryotic and eukaryotic) substrate specificity needs to be elucidated in the future.

To evaluate the possible biological function and engineering potential of HpDGAT2s to modulate TAG biosynthesis in algae and plants, in the present study, we generated heterologous expression lines in the evolutionarily close green alga *C. reinhardtii* CC849 and the model plant *A. thaliana*. It is not surprising that *HpDGAT2D* heterologous expression enhanced TAG contents in both *C. reinhardtii* CC849 (by ~ 1.4-fold) and *A. thaliana* (by ~ 1.2-fold). Guo et al. (2017) indicated that the *CeDGAT1* gene can stimulate FA biosynthesis and enhance seed weight and oil content when expressed in *A. thaliana* and *B. napus* [21]. Compared to the control, under 1/4 N stress conditions, it was also worth noting that the TAG content was significantly increased in a 4-day batch culture for *HpDGAT2D* heterologous expression lines under the same stress conditions (Fig. 4b), possibly due to the high transcription level (Fig. 4d). Wei et al. (2017) detected that, under nitrogen-replete conditions*, NoDGAT1A* expression in *C. reinhardtii* UVM4 had no effect on TAG accumulation, while TAG enhancement was observed under nitrogen-depleted conditions [22]. However, Mao et al. (2019) declared that *CzDGAT1A* expression in the oleaginous alga *N. oceanica* resulted in a considerable increase (~ 2.8-fold) in TAG levels [20]. Consistent with the strong preference for MUFAs and PUFAs rather than SFAs in yeast cells, HpDGAT2D also showed a similar trend in *C. reinhardtii*. Specifically, HpDGAT2D first opted for C16:1, C18:1, C18:2n6 and C18:3n3 rather than C16:2, C16:3, C16:4, C18:3n6, and C18:4n3. Interestingly, these preferred substrates were enriched in *C. reinhardtii*, indicating their potential for the engineering of *C. reinhardtii* for MUFAs- and PUFAs-enriched TAG production. This trend was also consistent with results from yeast cells in feeding tests (Fig. 3d and e) and consistent with previous studies of *NoDGAT1A* expression in *C. reinhardtii* UVM4 and *CzDGAT1A* expression in oleaginous alga *N. oceanica* by Wei et al. (2017) and Mao et al. (2019), respectively [20, 22]. In higher plants, the expression of *DGATs* generally enhances oil deposition in developing seeds [56]. For example, stronger expression of *DGAT1* was detected in developing seeds than in other tissues in soybeans [57]. However, *DGAT1* transcripts were also present in other plant tissues, although they were strongest in developing embryos and flower petals [58]. In the current study, the *HpDGAT2D* transcript was heterologously expressed in transgenic lines at different tissue organs, including roots, tubers, leaves, siliques, and seeds, to different extents (Fig. 5b). However, the exact process of FA change was much more complicated than those in yeast and *C. reinhardtii* cells (Fig. 5c). HpDGAT2D showed a strong preference for C18:1, C18:2n6, and C18:3n3 rather than C20:1, C20:2 and C22:1 in TAG biosynthesis, which was largely in agreement with the preference in yeast cells (Fig. 3d and e) and *C. reinhardtii* cells (Fig. 4c). Previous studies have indicated that seed-specific overexpression of *EgDGAT2* in *A. thaliana* enhanced the content of PUFAs C18:2n6 and C18:3n3 in seed TAG when compared to that from wild-type *Arabidopsis*. In turn, the proportion of C18:0 and C20:0 SFAs in seed TAG from *EgDGAT2* transgenic lines decreased accordingly [59]. In *Thraustochytrium aureum*, *DGAT2* expression under a strong seed-specific promoter in wild-type *A. thaliana* increased C18:2n6 content [60]. In addition, transgenic plants showed no other phenotypic differences. Therefore, HpDGAT2D should have great potential for increasing the specific oil production in other oil crops.

Although it has been previously suggested that DGATs may be involved in the esterification of AST in *H. lacustris* [34], there is no direct biochemical evidence to support this hypothesis. Recently, all 10 *CzDGATs* were expressed in a reconstructed AST-producing yeast strain [61] to examine whether these enzymes were responsible for EAST biosynthesis. However, no EAST was detected, indicating the null function of CzDGATs in AST esterification [20]. Considering the differences in genetic traits and AST biosynthetic pathways of both AST-producing algal strains, *C. zofingiensis* and *H. lacustris*, we will study the possible roles of HpDGAT2s in AST esterification in the future.

## Conclusions

Here, we performed an in-depth characterization of HpDGAT2s by integrating expression patterns, AST/TAG accumulation, functional complementation, and heterologous expression in yeast, plants, and algae. Five putative *DGAT2s* genes (*HpDGAT2A*, *HpDGAT2B*, *HpDGAT2C*, *HpDGAT2D*, and *HpDGAT2E*) were identified in *H. lacustris* by BLAST and CD analysis. These *DGAT2s* genes showed markedly increased transcription levels under stress conditions, which led to significant TAG and EAST accumulation. Functional complementation demonstrated that HpDGAT2A, HpDGAT2B, HpDGAT2D, and HpDGAT2E had the ability to restore TAG synthesis in a TAG-deficient yeast strain (H1246) with a large difference in enzymatic activity. FA profile assays revealed that HpDGAT2A, HpDGAT2D, and HpDGAT2E, but not HpDGAT2B, preferred MUFAs for TAG synthesis in yeast cells and showed PUFAs preference by feeding strategy. The heterologous expression of *HpDGAT2D* in wild-type *A. thaliana* and *C. reinhardtii* significantly increased the TAG content and showed a strong preference for MUFAs and PUFAs, indicating the engineering potential to increase specific TAG production in plants and algae.

## Methods

### Algal strain and growth conditions

The unicellular algal *Haematococcus lacustris/pluvialis* (FACHB-712) strain was obtained from the Freshwater Algae Culture Collection at the Institute of Hydrobiology and maintained at the Institute of Molecular Agriculture and Bioenergy (IMAB), Shanxi Agricultural University. *H. lacustris* was cultivated in 100-mL of BBM medium in 250-mL Erlenmeyer flasks. These Erlenmeyer flasks were placed under culture conditions of 25 μmol/m^2^/s light intensity with a diurnal cycle of 12 h light/12 h dark at 23 ± 1 °C. The culture solution was shaken for a fixed time twice a day. For the HL treatment, after the cultures were dark-adapted for 48 h, the later exponentially growing cultures (biomass content of approximately 200 mg/L) were further transferred into fresh medium under continuous white light (390–770 nm) or blue light (420–500 nm) with a light intensity of 500 μmol/m^2^/s without a light/dark cycle. For the 1/4 N nitrogen deficient treatment, the pre-cultured and dark-induced cells were collected and washed with nitrogen-free BBM medium and then further transferred into fresh BBM medium with 1/4 nitrogen content (the same as that in the BBM medium) under control culture conditions without a light/dark cycle. For HL and 1/4 N double stress (HL-1/4 N) treatment, pre-cultured and dark-induced cells were transferred into fresh medium with 1/4 nitrogen content under the same continuous white light or blue light. The cultures under control conditions were used as the control samples. These pre-cultured and dark-induced cells, after centrifugation and washing with sterilized water, were sampled as the starting point (N-0 day). The cultures were sampled N-1, N-2, N-3, and N-4 days after treatment. The cells were harvested by centrifugation (13,100 g at 4 °C for 5 min) and washed with PBS prior to storage in liquid nitrogen. For cell dry biomass determination, 20 mL of cell culture was collected and washed three times, and then the EP tubes containing cells were dried in a DW3 freeze-drier (Heto Dry Winner, Denmark).

### Cloning and bioinformatics analysis of HpDGAT2s

The genes encoding putative HpDGAT2s were predicted and cloned as follows: (1) the local BLAST program was used to predict *DGAT2s* genes based on the *H. lacustris* transcriptome database with annotated CzDGAT2s and CrDGAT2s (Additional file 1: Table S1), (2) the rapid amplification of cDNA ends (RACEs) method was used to obtain the full-length mRNA sequences and then determine their transcription start sites, stop sites, and encoding sequences, and (3) the open reading frame (ORF) for each *HpDGAT2s* gene was obtained by PCR again to construct distinct expression plasmids. All the primers used in this study are listed in Additional file 4: Table S4. The molecular weight (Mw), isoelectronic point (pI), subcellular localization, signal peptides (SP), chloroplast transfer peptides (CTP), transmembrane regions (TM), and phosphorylation site (Phos) of HpDGAT2s were predicted by Compute pI/MW, TargetP, ChloroP, SignalP, TMHMM, and NetPhos tools, respectively, in ExPASy [62]. HpDGAT2s and other DGATs from plants and algae were aligned using ClustalX [63]. Maximum likelihood trees of HpDGAT2s and other DGAT proteins were constructed using PhyML with the bootstrap (BS) values inferred from 400 replicates [64, 65]. Graphical representation and editing of the phylogenetic tree were performed with MEGA5 [66] and TreeDyn (v198.3) [67].

### RNA isolation and quantitative real-time PCR

Total RNA was extracted according to the EasySpin RNA Extraction Kit (Aidlab Biotech, Beijing, China) and was quantified by a NanoDrop 2000c (Thermo Scientific, USA). The first-strand cDNAs were synthesized according to the instruction of PrimeScript® RT Enzyme Mix I (TaKaRa DRR047A, China) Kit. The qRT-PCR was performed as described by our previous study using a 7500 Fast Real-Time PCR System (Applied Biosystems, Waltham, MA, USA) with SYBR Green PCR Master Mix (Invitrogen) [50]. The mRNA expression level was normalized using the *actin* gene as the internal control. All analyses were based on the CT values of the PCR products. The comparative CT method was used to investigate the transcriptional expression levels of *HpDGAT2s* genes [68].

### Functional complementation of HpDGAT2s in the TAG-deficient yeast H1246

The ORFs of *HpDGAT2A*, *HpDGAT2B*, *HpDGAT2D*, and *HpDGAT2E* were PCR-amplified using cDNA as a template and cloned into the yeast expression vector pYES2.0 (Invitrogen). After confirmation by restriction enzyme digestion and sequencing, the recombinant pYES2.0-*HpDGAT2s* plasmids were transformed into the *S. cerevisiae* TAG-producing strain INVSc1 or TAG-deficient quadruple mutant strain H1246 with the S.c. EasyComp Transformation Kit (Invitrogen) [20]. The expression of *HpDGAT2* genes in the yeast strain was verified at the transcript level by qRT-PCR. For the feeding experiments, yeast cultures were induced as described above but in the presence of 1% (w/v) Tergitol NP-40 (Sigma Aldrich, St. Louis, MO, USA) in the medium. At the beginning of induction, the appropriate FAs (C18:2n6, C18:3n3, C18:3n6, and C18:4n3) were added to the culture to a final concentration of 100 μM. Samples at an OD600 of 2.5 were harvested for lipid extraction, separation by TLC and analysis by GC.

### Heterologous expression of *HpDGAT2D* in *C. reinhardtii*

The nuclear transformation expression vector pDB124 (Additional file 9: Fig. S5), characterized in *C. reinhardtii* CC849 and gifted by professor Zhangli Hu from Shenzhen University [69], was used in this study after modification. The codon preference (*HpDGAT2D*) was optimized according to the alga *C. reinhardtii* (Additional file 10: Fig. S6) before constructing the expression vector. The codon preference optimized coding sequence of *HpDGAT2D* was amplified and cloned into the *Pml*I and *Bmt*I sites of pDB-124, followed by sequencing for verification. The resulting plasmid was linearized by *Xba*I and transformed into the *C. reinhardtii* cc849 strain via the glass beads method [70]. Transformants were selected on Tris-acetate-phosphate (TAP) plates with 10 μg/mL bleomycin (Sigma-Aldrich). For ND stress, the later exponentially growing *C. reinhardtii* cc849 cells (biomass content of approximately 420 mg/L) were used following the methods described in the above section. The integration of *HpDGAT2D* into the *Chlamydomonas* genome was verified by genomic PCR, and its transcription and protein expression levels were determined by qRT-PCR and western blotting using his-tagged antibodies, respectively. Considering that HpDGAT2D was a transmembrane protein, soluble and membrane proteins from *HpDGAT2D-His* fusion-heterologous expressing *C. reinhardtii* cells were used for immunodetection as previously described [19].

### Heterologous expression of *HpDGAT2D* in *A. thaliana*

The coding sequence of *HpDGAT2D* was amplified and cloned into *Eco*RI/*Xba*I sites of pCAMBIA1303 to yield pCAMBIA1303-*HpDGAT2D*. After verified by restriction enzyme digestion and sequencing, the pCAMBIA1303-*HpDGAT2D* vector was firstly transferred into *Agrobacterium tumefaciens* strain GV3101 [71], and finally transferred into *A. thaliana* plants by vacuum infiltration [72]. T1 generation seeds were selected on hygromycin (50 mg/L) and T2 transgenic *A. thaliana* lines were used for further analyses. The stable integration of pCAMBIA1301-*HpDGAT2D* into the genome and the transcription expression were determined by genomic PCR and qRT-PCR, respectively.

### Total astaxanthin analysis

The HPLC method was applied to quantify the contents of different AST forms using the standard curve of AST (purchased from Sigma-Aldrich) at known concentrations [50, 73].

### Lipid extraction and fatty acid analysis

Total lipids extraction, TAGs separation, and FAs analysis were performed according to previously described procedures [21, 74–76]. Briefly, 50 mg of yeast cells, 10 mg of freeze-dried algae cells or 10 mg of dried seeds were used to extract total lipids according to previously reported methods [75]. Then, TAGs were separated by thin-layer chromatography (TLC) methods as descripted in previous study [21]. Finally, TAGs were trans-esterified with 5% H_2_SO_4_ in methanol at 85 °C for 1 h and the fatty acid methyl esters (FAMEs) were analyzed by an Agilent GC equipped with a flame ionization detector (FID) and a capillary column (HP-88100 m × 0.25 mm × 0.2 mm) with an appropriate add amount of C17:0 FAME (Sigma) as an internal standard [75].

### Statistical analysis

All experiments were repeated three times to ensure reproducibility. The data were obtained as the mean value ± SD. Statistical analyses were performed using the SPSS statistical package (SPSS Inc., Chicago, IL, USA). Significant differences between treatments were statistically analyzed by paired-samples t-test. Statistical significance was achieved when *P* < 0.01.

## Supplementary Information

**Additional file 1 Table S1.** GenBank accession numbers (from National Center for Biotechnology Information) for DGAT and ACTIN proteins sequence used in this study. Note: Cz, *Chromochloris zofingiensis*; No, *Nannochloropsis oceanica*; Cr, *Chlamydomonas reinhardtii*; Pt, *Phaeodactylum tricornutum*; Li, *Lobosphaera incise*; At, *Arabidopsis thaliana*; Gm, *Glycine max*; Zm, *Zea mays*; Sc, *Saccharomyces cerevisiae*.

**Additional file 2 Table S2.** Gene sequences information and biochemical features of *HpDGAT2s* in *Haematococcus lacustris*. Note: ^a^Information regarding *HpDGAT2C* is predicted based on the partial coding sequence obtained from the transcriptome database.

**Additional file 3 Table S3.** Amino acid sequence identity (%; blue) and similarity (%; red) between HpDGAT2s and CrDGAT2s.

**Additional file 4 Table S4.** Primers used in this study.

**Additional file 5 Figure S1.** Predicated trans-membrane domains for HpDGAT2A, HpDGAT2B, HpDGAT2C, HpDGAT2D, and HpDGAT2E by TMHMM v. 2.0 Server (http://www.cbs.dtu.dk/services/TMHMM-2.0/).

**Additional file 6 Figure S2.** Predicated phosphorylation site for HpDGAT2A, HpDGAT2B, HpDGAT2C, HpDGAT2D, and HpDGAT2E by NetPhos 3.1 Server (http://www.cbs.dtu.dk/services/NetPhos/).

**Additional file 7 Figure S3.** Proteins sequences alignment of putative HpDGAT2s and other annotated DGAT2s from plants and microalgae. Proteins sequences with accession number used in this study were listed in in Additional file 1: Table S1. Blue color indicates the key conserved domains and black asterisk indicates the key amino acid residues.

**Additional file 8 Figure S4.** Phylogenetic analysis of HpDGAT2s and other annotated DGATs from higher plants and microalgae. Protein sequences used in this study were listed in Additional file 1: Table S1.

**Additional file 9 Figure S5.** A schematic map of the pDB124-*HpDGAT2D* vector. It contained an expression cassette of the *HpDGAT2D* gene under the control of the endogenous and characterized *PsaD* promoter and *PsaD* terminator, an expression cassette of the *Ble* gene controlled by the endogenous and characterized *RBCS2* promoter and *RBCS2* terminator, and an expression cassette of the *Amp* resistance gene, which conferred resistance to ampicillin.

**Additional file 10 Figure S6.** Original sequences of *HpDGAT2D* in *Haematococcus lacustris* and new sequences of *HpDGAT2D-Cr* after codon optimization for *Chlamydomonas reinhardtii*. Red color stands for the modified nucleotide sequence.

**Additional file 11 Figure S7**. Genomic level of *HpDGAT2D* in *C. reinhardtii* cells (a) and western blotting of HpDGAT2D-6-His tag fusion protein with His-tag antibody (b). Note: Soluble and membrane proteins were separated and used for blotting. Actin which was known soluble protein was used as controls. M, DNA marker or protein marker (Non-western blotting type).

## Data Availability

The datasets supporting the results of this article are included within the article and its additional files. The five DGATs proteins (*HpDGAT2A*: MT875161; *HpDGAT2B*: MT875162; *HpDGAT2C*: MT875163; *HpDGAT2D*: MT875164; *HpDGAT2E*: MT875165) from *Haematococcus lacustris* have been deposited in NCBI database. All the other raw data of protein sequences used in the current study were downloaded from the NCBI database (https://www.ncbi.nlm.nih.gov/protein/?term=dgat) and the GenBank accession numbers were listed in Additional file 1: Table S1.

## Abbreviations

AST: Astaxanthin
E-AST: Esterified astaxanthin
F-AST: Free AST
M-AST: Momoester AST
D-AST: Diester AST
TAG: Triacylglycerol
DGAT: Diacylglycerol acyltransferase
ORF: Open reading frame
RACEs: Rapid amplification of complementary DNA ends
UTR: Untranslated region
HL: High light
ND: Nitrogen deficient
MUFAs: Monounsaturated fatty acyl-CoAs
PUFAs: Polyunsaturated fatty acyl-CoAs
