# Supplementary material for: Characterization of type-2 diacylglycerol acyltransferases in Haematococcus lacustris reveals their functions and engineering potential in triacylglycerol biosynthesis
Source: BMC Plant Biol. 2021 Jan 6;21:20. doi: 10.1186/s12870-020-02794-6 (PMC7788937; doi:10.1186/s12870-020-02794-6)
Supplement: Supplementary file 1 — Additional file 1 Table S1. GenBank accession numbers (from National Center for Biotechnology Information) for DGAT and ACTIN proteins sequence used in this study. Note: Cz, Chromochloris zofingiensis; No, Nannochloropsis oceanica; Cr, Chlamydomonas reinhardtii; Pt, Phaeodactylum tricornutum; Li, Lobosphaera incise; At, Arabidopsis thaliana; Gm, Glycine max; Zm, Zea mays; Sc, Saccharomyces cerevisiae. [file 12870_2020_2794_MOESM1_ESM.pdf]

Additional file 1: Table S1 GenBank accession numbers (from National Center for Biotechnology Information) for DGAT and ACTIN proteins sequence used in this study.

Cz, *Chromochloris zofingiensis*; No, *Nannochloropsis oceanica*; Cr, *Chlamydomonas reinhardtii*; Pt, *Phaeodactylum tricornutum*; Li, *Lobosphaera incise*; At, *Arabidopsis thaliana*; Gm, *Glycine max*; Zm, *Zea mays*; Sc, *Saccharomyces cerevisiae*.

Gene	GenBank No.	Gene	GenBank No.
CzDGAT1A	QBG05553.1	*CrDGAT1*	XP_001692975.1
CzDGAT1B	QBG05554.1	*CrDGAT2A*	AGO32156.1
CzDGAT2A	QBG05555.1	*CrDGAT2B*	AGO32157.1
CzDGAT2B	QBG05556.1	*CrDGAT2C*	AGO32158.1
CzDGAT2C	QBG05557.1	*CrDGAT2D*	AGO32159.1
CzDGAT2D	QBG05558.1	*CrDGAT2E*	XP_001701667.1
CzDGAT2E	QBG05559.1	*CrDGAT3*	XP_001691342.1
CzDGAT2F	QBG05560.1	*PtDGAT1*	ADY76581.1
CzDGAT2G	QBG05561.1	*PtDGAT2A*	AFQ23659.1
CzDGAT2H	QBG05562.1	*PtDGAT2B*	AFM37314.1
NoDGAT1A	ASL69957.1	*PtDGAT2C*	AFQ23660.1
NoDGAT1B	ASL69958.1	*PtDGAT2D*	AFQ23661.1
NoDGAT2A	ATB53137.1	*PtWSD*	XP_002180007.1
NoDGAT2B	ATB53138.1	*AtDGAT1*	CAB45373.1
NoDGAT2C	ATB53139.1	*AtDGAT2*	NP_566952.1
NoDGAT2D	ATB53140.1	*GmDGAT3*	XP_003542403.1
NoDGAT2E	ATB53141.1	*GmWSD*	XP_003552517.1
NoDGAT2F	ATB53142.1	*LiDGAT1*	MF576159
NoDGAT2G	ATB53143.1	*LiDGAT2.1*	MH290880
NoDGAT2H	ATB53144.1	*LiDGAT2.2*	MH290881
NoDGAT2I	ATB53145.1	*LiDGAT2.3*	MH290882
NoDGAT2J	ATB53146.1	*ZmDGAT3*	PWZ45621.1
NoDGAT2K	ATB53136.1	*HpDGAT2A*	MT875161
HpDGAT2B	MT875162	*HpDGAT2C*	MT875163
HpDGAT2D	MT875164	*HpDGAT2E*	MT875165
ScACTIN	AAA34391.1	*CrACTIN*	D50838.1
AtACTIN	NP_190236.1		
