# Supplementary material for: Characterization of type-2 diacylglycerol acyltransferases in Haematococcus lacustris reveals their functions and engineering potential in triacylglycerol biosynthesis
Source: BMC Plant Biol. 2021 Jan 6;21:20. doi: 10.1186/s12870-020-02794-6 (PMC7788937; doi:10.1186/s12870-020-02794-6)
Supplement: Supplementary file 2 — Additional file 2 Table S2. Gene sequences information and biochemical features of HpDGAT2s in Haematococcus lacustris. Note: aInformation regarding HpDGAT2C is predicted based on the partial coding sequence obtained from the transcriptome database. [file 12870_2020_2794_MOESM2_ESM.pdf]

Additional file 2: Table S2 Gene sequences information and biochemical features of HpDGAT2s in *Haematococcus lacustris*.

[a]Information regarding *HpDGAT2C* is predicted based on the partial coding sequence obtained from the transcriptome database.

Gene	mRNA	ORF	5'-UTR	3'-UTR	PI	Mw	cTP	SignalP	TM	Phos
HpDGAT2A	1, 446	1, 149	247	50	9.23	41.9	-	-	2	27
HpDGAT2B	1, 193	1, 017	133	43	9.70	37.8	-	-	3	25
HpDGAT2C[a]	1, 730	1, 443	0	287	9.83	52.9	+	-	2	30
HpDGAT2D	1, 415	954	9	452	9.66	34.8	-	-	2	14
HpDGAT2E	1, 071	990	81	0	9.36	40.4	-	-	2	21
