# Supplementary material for: Characterization of type-2 diacylglycerol acyltransferases in Haematococcus lacustris reveals their functions and engineering potential in triacylglycerol biosynthesis
Source: BMC Plant Biol. 2021 Jan 6;21:20. doi: 10.1186/s12870-020-02794-6 (PMC7788937; doi:10.1186/s12870-020-02794-6)
Supplement: Supplementary file 3 — Additional file 3 Table S3. Amino acid sequence identity (%; blue) and similarity (%; red) between HpDGAT2s and CrDGAT2s. [file 12870_2020_2794_MOESM3_ESM.pdf]

Additional file 3: Table S3 Amino acid sequence identity (%; blue) and similarity (%; red) between HpDGAT2s and CrDGAT2s.

Gene	*HpDGAT2A*	*HpDGAT2B*	*HpDGAT2C*	*HpDGAT2D*	*HpDGAT2E*	*CrDGAT2A*	*CrDGAT2B*	*CrDGAT2C*	*CrDGAT2D*	*CrDGAT2E*
HpDGAT2A	100	32	29	25	48	40	31	27	31	27
HpDGAT2B	42	100	33	32	28	28	54	48	36	39
HpDGAT2C	45	28	100	30	28	26	28	29	29	32
HpDGAT2D	32	33	33	100	33	30	31	31	49	30
HpDGAT2E	41	48	46	50	100	27	51	59	30	40
CrDGAT2A	55	44	40	47	40	100	29	30	33	28
CrDGAT2B	46	71	43	48	71	44	100	53	32	42
CrDGAT2C	43	68	45	46	73	45	70	100	30	41
CrDGAT2D	44	50	42	65	49	51	47	48	100	34
CrDGAT2E	42	58	47	46	59	46	63	60	53	100
