# Supplementary material for: Characterization of type-2 diacylglycerol acyltransferases in Haematococcus lacustris reveals their functions and engineering potential in triacylglycerol biosynthesis
Source: BMC Plant Biol. 2021 Jan 6;21:20. doi: 10.1186/s12870-020-02794-6 (PMC7788937; doi:10.1186/s12870-020-02794-6)
Supplement: Supplementary file 4 — Additional file 4 Table S4. Primers used in this study. [file 12870_2020_2794_MOESM4_ESM.pdf]

Additional file 4: Table S4 Primers used in this study.

Names	Forward primer (5'–3')	Reverse primer (5'–3')	Note
HpDGAT2A-5-R1		ATGCCTGGGTACAACTTGGAGAA	5'-RACE
HpDGAT2A-5-R2		GGTCCAGGTCCGCTGTCTTGT	5'-RACE
HpDGAT2A-3-F1	TCCTGCCTCAACCTGCTCACA		3'-RACE
HpDGAT2A-3-F2	GCATCATGCGGGTGGCACTAC		3'-RACE
HpDGAT2B-5-R1		AGGGAACTCAGCCATCACCAA	5'-RACE
HpDGAT2B-5-R2		AGGGTGGACAACAGCACAATCAA	5'-RACE
HpDGAT2B-3-F1	AGCAAGGGCTCCGTGGCGGTGATA		3'-RACE
HpDGAT2B-3-F2	CACCTATCCAAATTGCGAAGACG		3'-RACE
HpDGAT2C-5-R1		TGCTGACACCACCACCACCACC	5'-RACE
HpDGAT2C-5-R2		TGCATCTGCCAGCGCCGCCATT	5'-RACE
HpDGAT2C-3-F1	GCCTGTGGGTCCGTTCTTTGAGC		3'-RACE
HpDGAT2C-3-F2	CGCCGATGACGCCTACGATGT		3'-RACE
HpDGAT2D-5-R1		CGAAGGGCACCGCAAAGCACA	5'-RACE
HpDGAT2D-5-R2		AAGCCATCCTCATCTTCCACAACTA	5'-RACE
HpDGAT2D-3-F1	TTCGCTGGATTAGCAGAAAGGTG		3'-RACE
HpDGAT2D-3-F2	TACTTGGACCGCTTCATACACGACA		3'-RACE
HpDGAT2E-5-R1		GAACACCCACAGCTTGCAGAAT	5'-RACE
HpDGAT2E-5-R2		GCCACGCCTAGCATAATGAGC	5'-RACE
HpDGAT2E-3-F1	AGATGTACCTGGTTCATGGCAA		3'-RACE
HpDGAT2E-3-F2	GCCAAGGACGACCCTGAGTTT		3'-RACE
HpDGAT2A-ORF-F/R	ATGCGCAAGTTTGCACAGACGA	TCACTGCACAAACTCCAGGCTC	ORF clone
HpDGAT2B-ORF-F/R	ATGGGTGTCGCAACGAATGCGAC	TCACTGGATCTCCAGCGGCTTGTCC	ORF clone
HpDGAT2D-ORF-F/R	ATGCCGGCCTTGCCTAGGCCA	CTACAAGATGCGCAACTCGCAT	ORF clone
HpDGAT2D-Cr-ORF-F/R	ATGCCCGCCCTGCCCCGCCCCCT	TTACAGGATGCGCAGCTCGCAGCC	ORF clone
HpDGAT2E-ORF-F/R	ATGGGCGTTAAAAAGCCAGTCTTCG	TCACTCGATGCTCAGCGGCTTCTCA	ORF clone
HpDGAT2A-Q-F/R	TCCTGCCTCAACCTGCTCACA	TGGGCTCTGCCACGTCAAACA	qRT-PCR
HpDGAT2B-Q-F/R	AGGGCTCCGTGGCGGTGATAGT	CGTCTTCGCAATTTGGATAGGTG	qRT-PCR
HpDGAT2C-Q-F/R	TGGAGGGCAAGTTTGACGAGTGG	TGCTGCGAAGGGCACGATGAG	qRT-PCR
HpDGAT2D-Q-F/R	CTCATTGGGCTCGTGCTGTTG	TCAAGCCATCCTCATCTTCCACA	qRT-PCR
HpDGAT2E-Q-F/R	CAGCCAACTCTGTCTTTAACATTCC	GCGGTGGTTCTCATACAGGTCTT	qRT-PCR
HpDGAT2A-YES-F/R	gaattcGATGCGCAAGTTTGCACAGACG	agatctTCACTGCACAAACTCCAGGCT	Yeast
HpDGAT2B-YES-F/R	gaattcGATGGGTGTCGCAACGAATGCGA	agatctTCACTGGATCTCCAGCGGCTT	Yeast
HpDGAT2D-YES-F/R	gaattcGATGCCGGCCTTGCCTAGGCCA	agatctCTACAAGATGCGCAACTCGCA	Yeast
HpDGAT2E-YES-F/R	gaattcGATGGGCGTTAAAAAGCCAGTCT	agatctTCACTCGATGCTCAGCGGCTT	Yeast
ScACTIN-Q-F/R	ACGTCGCCTTGGACTTCGAA	AGATGGAGCCAAAGCGGTGA	qRT-PCR
HpDGAT2D-DB-F/R	cacgtgATGCCGGCCTTGCCTAGGCCA	cgatcgCTACAAGATGCGCAACTCGCA	Algae
HpDGAT2D-Cr-Q-F/R	TGGCCAGCAGCGTGTGCTTCG	CACGAAGCCGTGGCGCTTGC	Algae
CrACTIN-Q-F/R	GATGCCACGGGCGATGATTGA	CGTTGAGTACCCTCCCTGCGTTG	qRT-PCR
HpDGAT2D-1303-F/R	gaattcGTGCCGGCCTTGCCTAGGCCA	agatctCTACAAGATGCGCAACTCGCA	Plants
AtACTIN-Q-F/R	ATGACATGGAGAAGATCTGGCATCA	AGCCTGGATGGCAACATACATAGC	qRT-PCR

Sequences in lower-case letters indicate enzyme restriction sites.
