# Supplementary material for: Characterization of type-2 diacylglycerol acyltransferases in Haematococcus lacustris reveals their functions and engineering potential in triacylglycerol biosynthesis
Source: BMC Plant Biol. 2021 Jan 6;21:20. doi: 10.1186/s12870-020-02794-6 (PMC7788937; doi:10.1186/s12870-020-02794-6)
Supplement: Supplementary file 5 — Additional file 5 Figure S1. Predicated trans-membrane domains for HpDGAT2A, HpDGAT2B, HpDGAT2C, HpDGAT2D, and HpDGAT2E by TMHMM v. 2.0 Server (http://www.cbs.dtu.dk/services/TMHMM-2.0/). [file 12870_2020_2794_MOESM5_ESM.pdf]

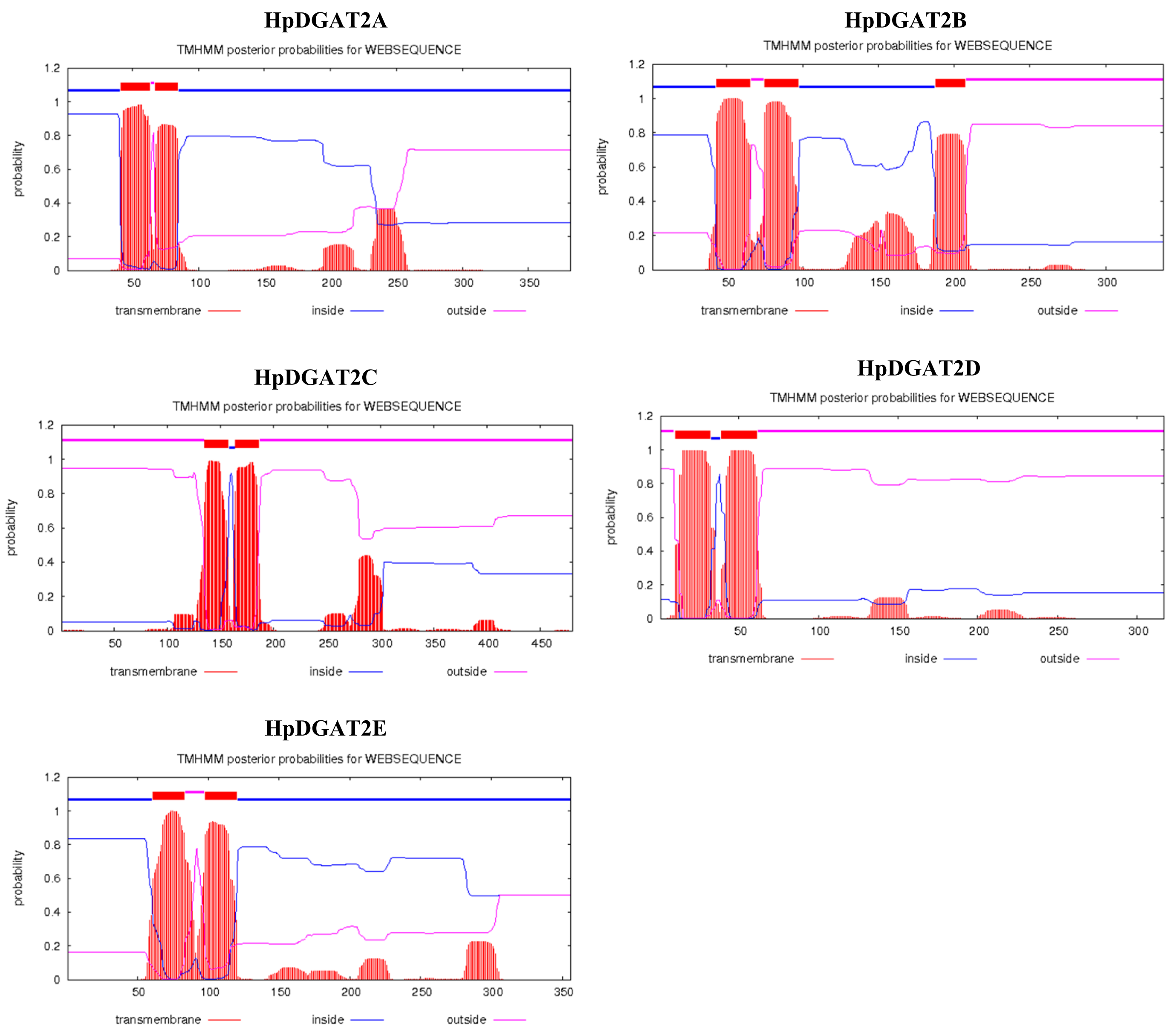

Additional file 5: Figure S1 Predicated trans-membrane domains for HpDGAT2A, HpDGAT2B, HpDGAT2C, HpDGAT2D, and HpDGAT2E by TMHMM v. 2.0 Server (http://www.cbs.dtu.dk/services/TMHMM-2.0/).
