# Supplementary material for: Characterization of type-2 diacylglycerol acyltransferases in Haematococcus lacustris reveals their functions and engineering potential in triacylglycerol biosynthesis
Source: BMC Plant Biol. 2021 Jan 6;21:20. doi: 10.1186/s12870-020-02794-6 (PMC7788937; doi:10.1186/s12870-020-02794-6)
Supplement: Supplementary file 6 — Additional file 6 Figure S2. Predicated phosphorylation site for HpDGAT2A, HpDGAT2B, HpDGAT2C, HpDGAT2D, and HpDGAT2E by NetPhos 3.1 Server (http://www.cbs.dtu.dk/services/NetPhos/). [file 12870_2020_2794_MOESM6_ESM.pdf]

HpDGAT2A

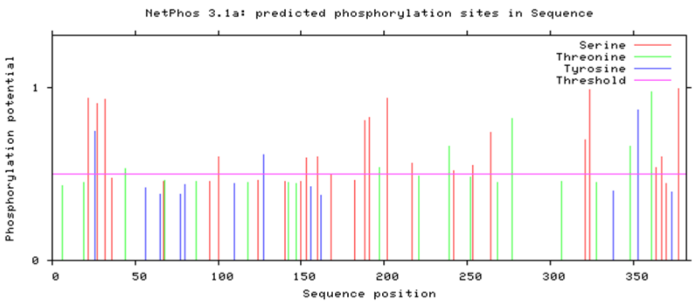

HpDGAT2B

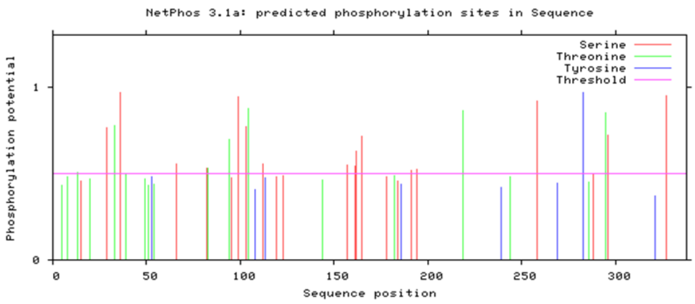

HpDGAT2C

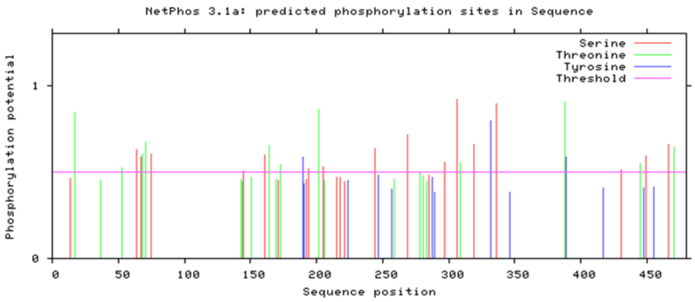

HpDGAT2D

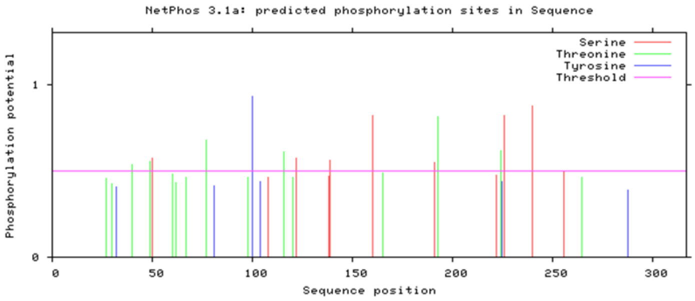

HpDGAT2E

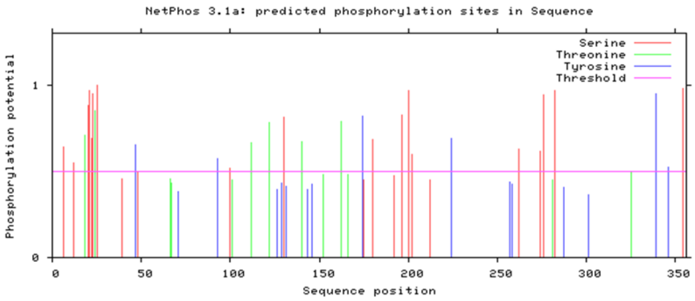

Additional file 6: Figure S2 Predicated phosphorylation site for HpDGAT2A, HpDGAT2B, HpDGAT2C, HpDGAT2D, and HpDGAT2E by NetPhos 3.1 Server (http://www.cbs.dtu.dk/services/NetPhos/).
