# Supplementary material for: Characterization of type-2 diacylglycerol acyltransferases in Haematococcus lacustris reveals their functions and engineering potential in triacylglycerol biosynthesis
Source: BMC Plant Biol. 2021 Jan 6;21:20. doi: 10.1186/s12870-020-02794-6 (PMC7788937; doi:10.1186/s12870-020-02794-6)
Supplement: Supplementary file 7 — Additional file 7 Figure S3. Proteins sequences alignment of putative HpDGAT2s and other annotated DGAT2s from plants and microalgae. Proteins sequences with accession number used in this study were listed in in Additional file 1: Table S1. Blue color indicates the key conserved domains and black asterisk indicates the key amino acid residues. [file 12870_2020_2794_MOESM7_ESM.pdf]

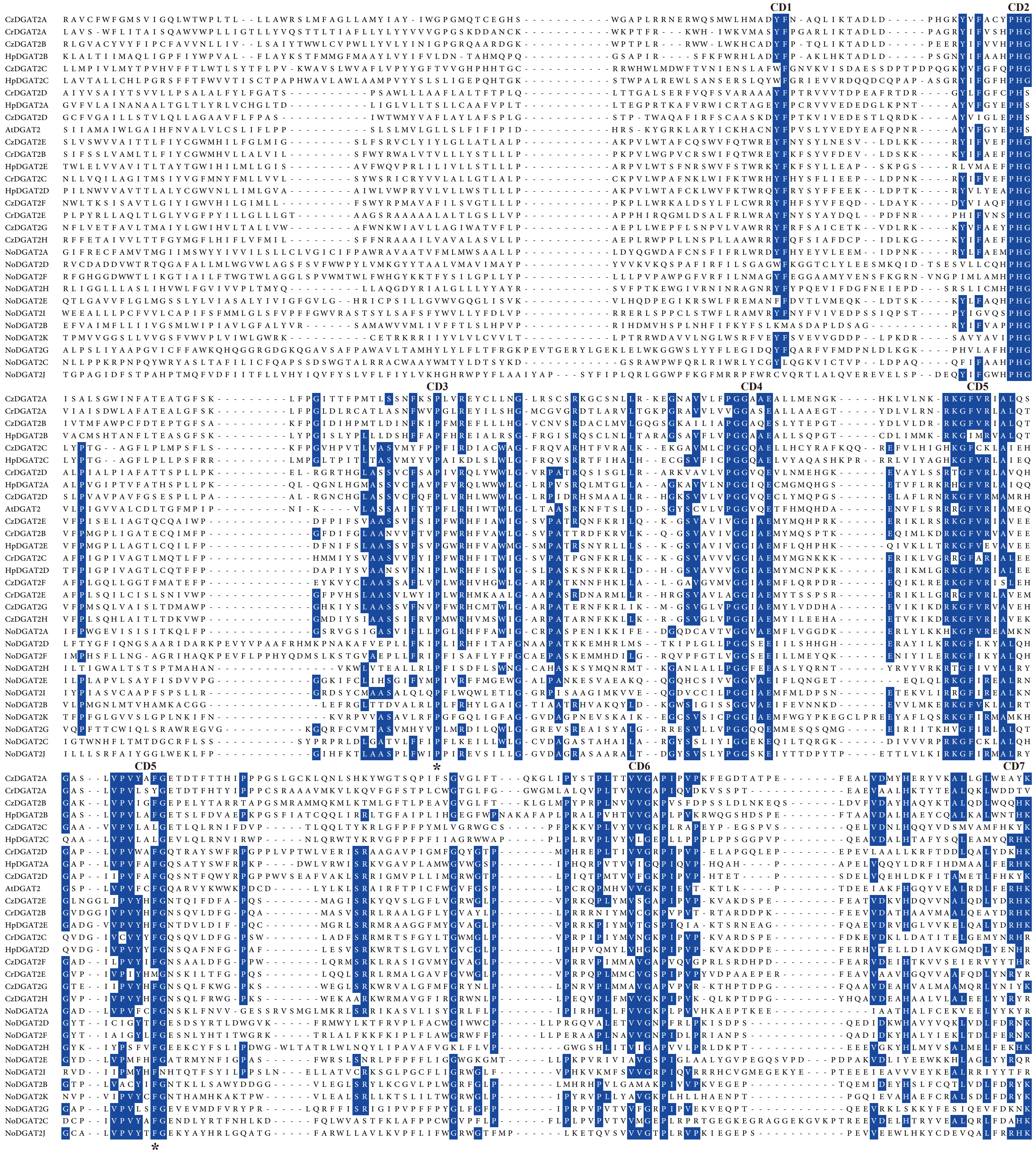

Additional file 7: Figure S3 Protein sequence alignment of putative HpDGAT2s and other annotated DGAT2s from plants and microalgae. Protein sequences used in this study were listed in in Additional file 1: Table S1. Blue color indicates the key conserved domains and black asterisk indicates the key amino acid residues.
