# Supplementary material for: Characterization of type-2 diacylglycerol acyltransferases in Haematococcus lacustris reveals their functions and engineering potential in triacylglycerol biosynthesis
Source: BMC Plant Biol. 2021 Jan 6;21:20. doi: 10.1186/s12870-020-02794-6 (PMC7788937; doi:10.1186/s12870-020-02794-6)
Supplement: Supplementary file 8 — Additional file 8 Figure S4. Phylogenetic analysis of HpDGAT2s and other annotated DGATs from higher plants and microalgae. Protein sequences used in this study were listed in Additional file 1: Table S1. [file 12870_2020_2794_MOESM8_ESM.pdf]

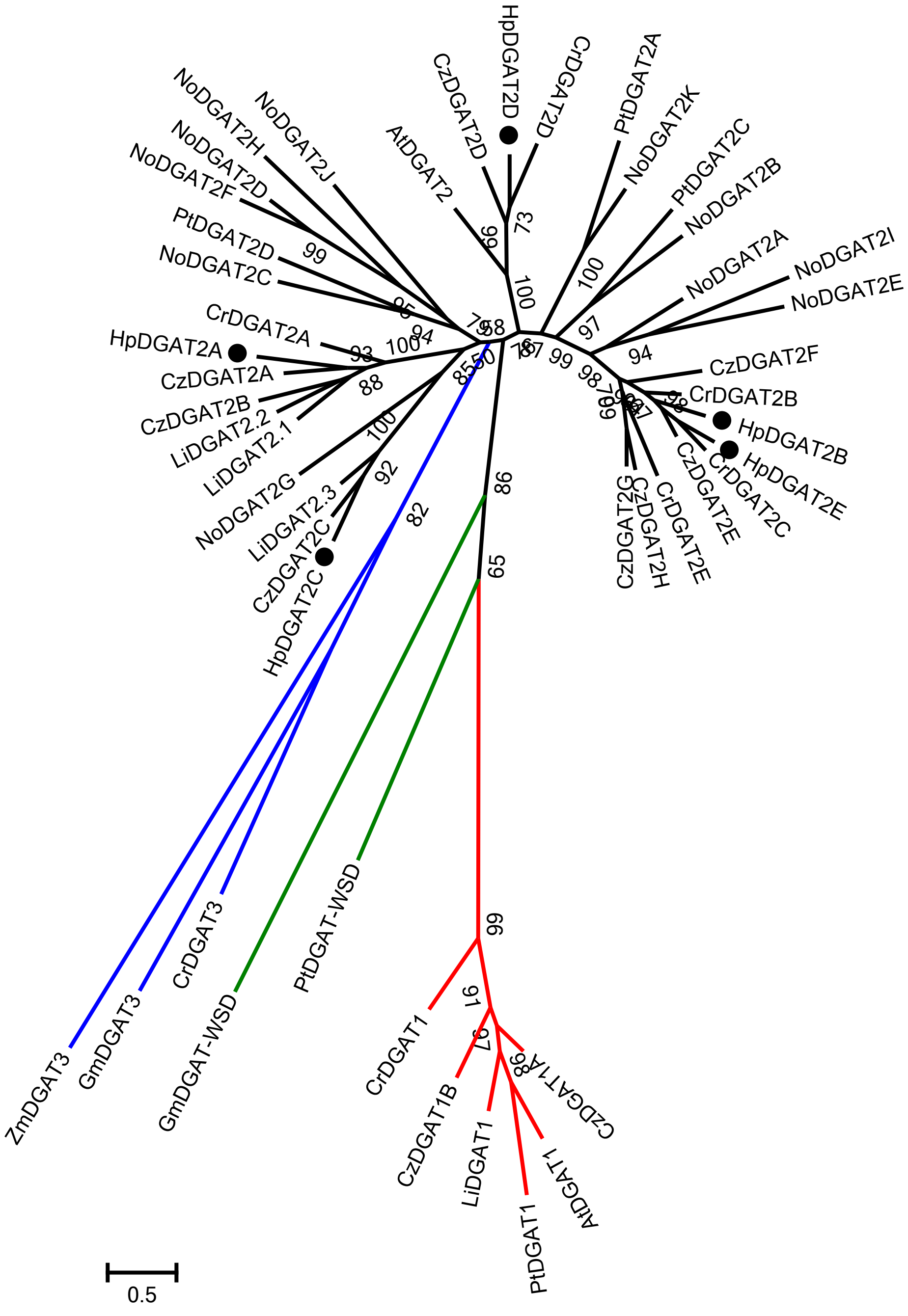

Additional file 8: Figure S4 Phylogenetic analysis of HpDGAT2s and other annotated DGATs from higher plants and microalgae. Protein sequences used in this study were listed in Additional file 1: Table S1.
