# Supplementary material for: Characterization of type-2 diacylglycerol acyltransferases in Haematococcus lacustris reveals their functions and engineering potential in triacylglycerol biosynthesis
Source: BMC Plant Biol. 2021 Jan 6;21:20. doi: 10.1186/s12870-020-02794-6 (PMC7788937; doi:10.1186/s12870-020-02794-6)
Supplement: Supplementary file 9 — Additional file 9 Figure S5. A schematic map of the pDB124-HpDGAT2D vector. It contained an expression cassette of the HpDGAT2D gene under the control of the endogenous and characterized PsaD promoter and PsaD terminator, an expression cassette of the Ble gene controlled by the endogenous and characterized RBCS2 promoter and RBCS2 terminator, and an expression cassette of the Amp resistance gene, which conferred resistance to ampicillin. [file 12870_2020_2794_MOESM9_ESM.pdf]

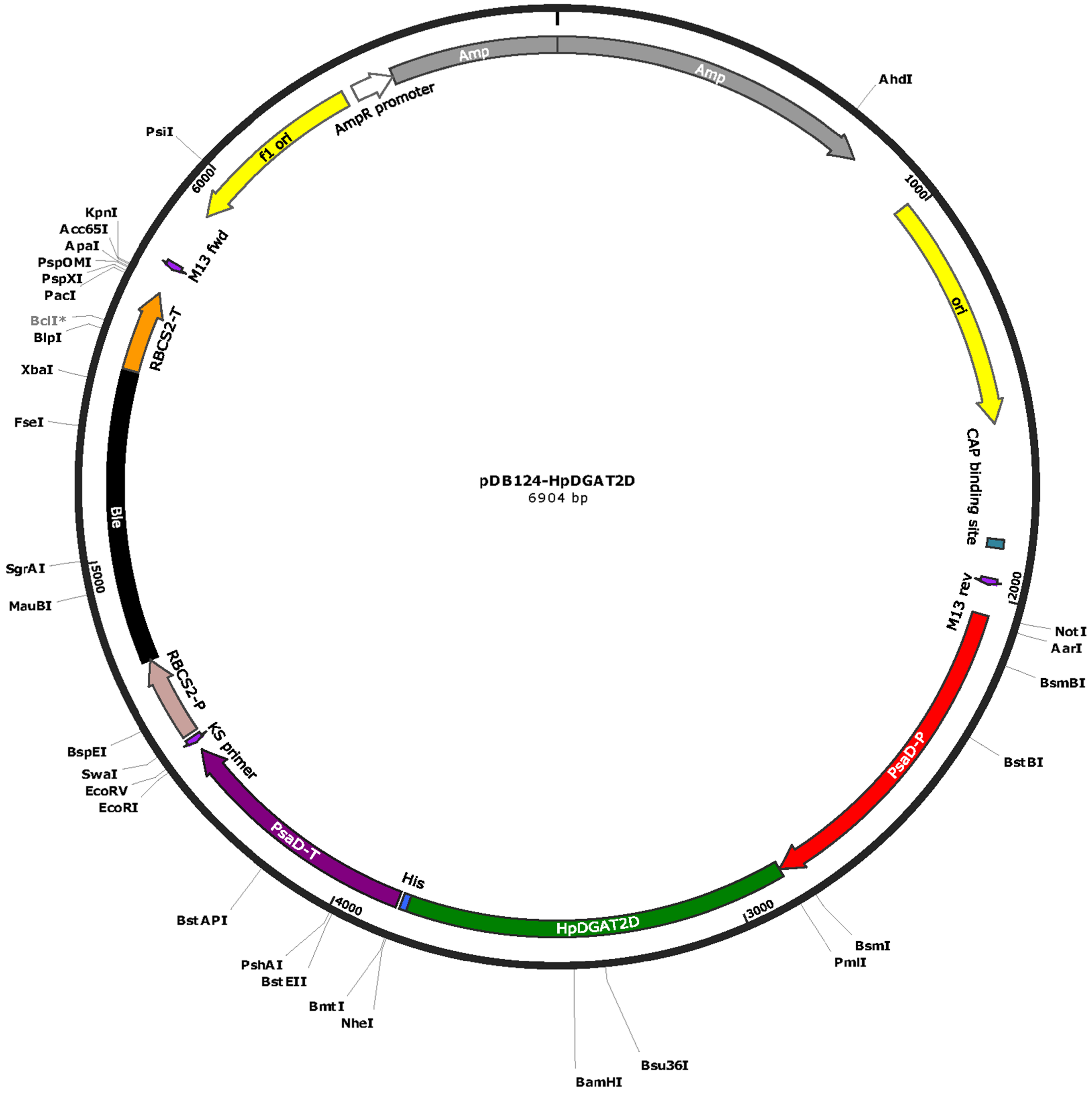

Additional file 9: Figure S5 A schematic map of the pDB124-HpDGAT2D plasmid. It contained an expression cassette of the *HpDGAT2D* gene under the control of the endogenous and characterized *PsaD* promoter and *PsaD* terminator, an expression cassette of the *Ble* gene controlled by the endogenous and characterized *RBCS2* promoter and *RBCS2* terminator, and an expression cassette of the *Amp* resistance gene, which conferred resistance to ampicillin.
