# Supplementary material for: Characterization of type-2 diacylglycerol acyltransferases in Haematococcus lacustris reveals their functions and engineering potential in triacylglycerol biosynthesis
Source: BMC Plant Biol. 2021 Jan 6;21:20. doi: 10.1186/s12870-020-02794-6 (PMC7788937; doi:10.1186/s12870-020-02794-6)
Supplement: Supplementary file 10 — Additional file 10 Figure S6. Original sequences of HpDGAT2D in Haematococcus lacustris and new sequences of HpDGAT2D-Cr after codon optimization for Chlamydomonas reinhardtii. Red color stands for the modified nucleotide sequence. [file 12870_2020_2794_MOESM10_ESM.pdf]

**>*HpDGAT2D* (original sequence)**

ATGCCGGCCTTGCCTAGGCCATTACGGGCTCTGCCGTTAGGGGTGTTTGTACTCGCAATCAACGCAAATGCGGCGCT
CACTGGGCTAACGCTGTACCGGCTTGTGTGCCACGGTCTGACGGACCTCATTGGGCTCGTGCTGTTGACATCGCTTT
TGTGCGCTGCTTTCGTCCCTTTGACGTTGACCGAGGGACCGCGGACAAAAGCGTTTGTAAGATGGATCTGCAGAACA
GCCGGCGAGTACTTTCCATGCCGTGTAGTTGTGGAAGATGAGGATGGCTTGAAGCCCAACACAGCCTATGTGTTTGG
ATATGAGCCGCACTCAGCTCTGCCCGTGGGCATACCAACTGTCTTTGCCACGCACTCCCCCCTGCTGCCAAAGCAGC
TGCAGGGCAACCTGCACGGCATGGCGTCCTCCGTGTGCTTTGCGGTGCCCTTCGTCCGGCAGCTGTGGTGGTGGCTA
GGCCTGAGGCCGGTGAGTCGGCAGCTGATGACTGGGCTGCTGGCGGCGGGCAAGGCCGTGGTCCTCAACCCCGGG
GGGATCCAGGAGTGCATGGGCATGCAACACGGCAGCGAGACAGTGTTCCTGCGCAAGCGCCATGGCTTTGTCCGCC
TGGCGATACAGCAGGGCGCACCCCTGGTGCCAGTCTTTGCTTTTGGTCAGAGTGCCACCTACAGCTGGTTTCGCCCC
AAGCCTGACTGGCTGGTTCGCTGGATTAGCAGAAAGGTGGGCGCCGTGCCCCTGGCCATGTGGGGGGTGTGGGGCT
CCCCCATTCCCCACCAGCGCCCTGTCACGGTGGTGATTGGGCAGCCCATCCAGGTCCCCCACCAGGCCCATCCCGCG
CCTGAGCTGGTGCAGCAGTACTTGGACCGCTTCATACACGACATGGCGGCGTTGTTTGAGCGACACAAGGCCGCGTG
TGGCCAGGCGGGATGCGAGTTGCGCATCTTGTAG

**>*HpDGAT2D-Cr* (new sequence after codon optimization for *Chlamydomonas reinhardtii*)**

ATGCCCGCCCTGCCCCGCCCCCTGCGCGCCCTGCCCCTGGGCGTGTTCGTGCTGGCCATCAACGCCAACGCCGCCCT
GACCGGCCTGACCCTGTACCGCCTGGTGTGCCACGGCCTGACCGACCTGATCGGCCTGGTGCTGCTGACCAGCCTGC
TGTGCGCCGCCTTCGTGCCCCTGACCCTGACCGAGGGCCCCCGCACCAAGGCCTTCGTGCGCTGGATCTGCCGCACC
GCCGGCGAGTACTTCCCCTGCCGCGTGGTGGTGGAGGACGAGGACGGCCTGAAGCCCAACACCGCCTACGTGTTCG
GCTACGAGCCCCACAGCGCCCTGCCCGTGGGCATCCCCACCGTGTTCGCCACCCACAGCCCCCTGCTGCCCAAGCAG
CTGCAGGGCAACCTGCACGGCATGGCCAGCAGCGTGTGCTTCGCCGTGCCCTTCGTGCGCCAGCTGTGGTGGTGGC
TGGGCCTGCGCCCCGTGAGCCGCCAGCTGATGACCGGCCTGCTGGCCGCCGGCAAGGCCGTGGTGCTGAACCCCGG
CGGCATCCAGGAGTGCATGGGCATGCAGCACGGCAGCGAGACCGTGTTCCTGCGCAAGCGCCACGGCTTCGTGCGC
CTGGCCATCCAGCAGGGCGCCCCCCTGGTGCCCGTGTTCGCCTTCGGCCAGAGCGCCACCTACAGCTGGTTCCGCCC
CAAGCCCGACTGGCTGGTGCGCTGGATCAGCCGCAAGGTGGGCGCCGTGCCCCTGGCCATGTGGGGCGTGTGGGGC
AGCCCCATCCCCCACCAGCGCCCCGTGACCGTGGTGATCGGCCAGCCCATCCAGGTGCCCCACCAGGCCCACCCCGC
CCCCGAGCTGGTGCAGCAGTACCTGGACCGCTTCATCCACGACATGGCCGCCCTGTTCGAGCGCCACAAGGCCGCCT
GCGGCCAGGCCGGCTGCGAGCTGCGCATCCTGTAA

**Additional file 10: Figure S6 Original sequence of *HpDGAT2D* in *Haematococcus lacustris* and new sequence of *HpDGAT2D-Cr* after codon optimization for *Chlamydomonas reinhardtii*. Red color stands for the modified nucleotide sequence.**
