# Supplementary material for: Characterization of type-2 diacylglycerol acyltransferases in Haematococcus lacustris reveals their functions and engineering potential in triacylglycerol biosynthesis
Source: BMC Plant Biol. 2021 Jan 6;21:20. doi: 10.1186/s12870-020-02794-6 (PMC7788937; doi:10.1186/s12870-020-02794-6)
Supplement: Supplementary file 11 — Additional file 11 Figure S7. Genomic level of HpDGAT2D in C. reinhardtii cells (a) and western blotting of HpDGAT2D-6-His tag fusion protein with His-tag antibody (b). Note: Soluble and membrane proteins were separated and used for blotting. Actin which was known soluble protein was used as controls. M, DNA marker or protein marker (Non-western blotting type). [file 12870_2020_2794_MOESM11_ESM.pdf]

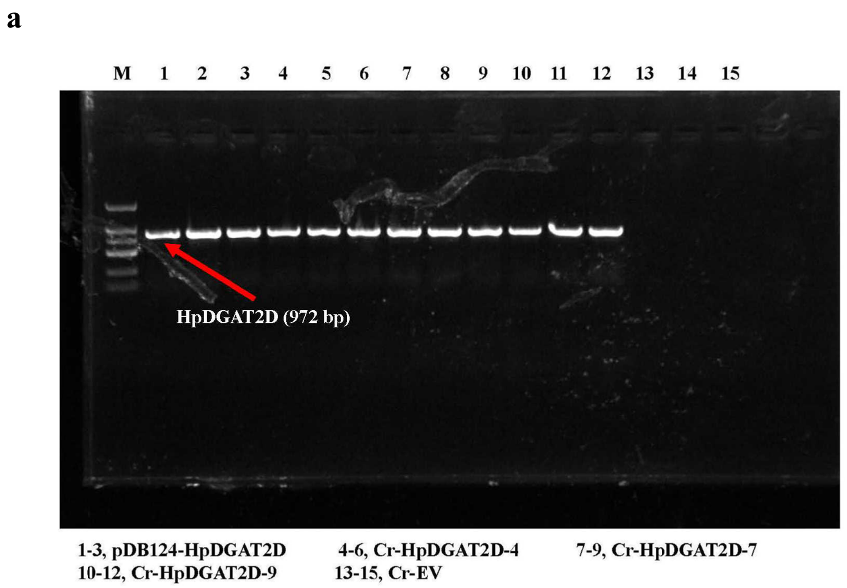

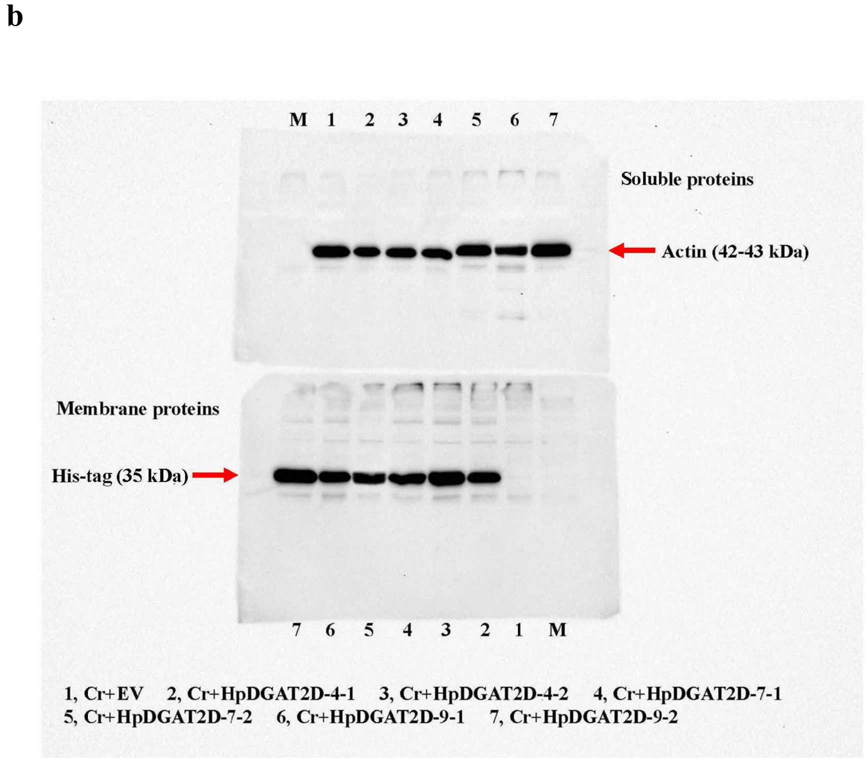

Additional file 11: Figure S7 Genomic level of *HpDGAT2D* in *C. reinhardtii* cells (a) and western blotting of HpDGAT2D-6-His tag fusion protein with His-tag antibody (b). Note: Soluble and membrane proteins were separated and used for blotting. Actin which was known soluble protein was used as controls. M, DNA marker or protein marker (Non-western blotting type).
